# Shared cross-cultural principles underlie human prosocial behavior at the smallest scale

**DOI:** 10.1038/s41598-023-30580-5

**Published:** 2023-04-19

**Authors:** Giovanni Rossi, Mark Dingemanse, Simeon Floyd, Julija Baranova, Joe Blythe, Kobin H. Kendrick, Jörg Zinken, N. J. Enfield

**Affiliations:** 1 Department of Sociology, UCLA, Los Angeles, USA; 2 Centre for Language Studies, Radboud University, Nijmegen, The Netherlands; 3 Department of Anthropology, Universidad San Francisco de Quito, Quito, Ecuador; 4 Department of Linguistics, Macquarie University, Sydney, Australia; 5 Department of Language and Linguistic Science, University of York, York, UK; 6 Leibniz Institute for the German Language, Mannheim, Germany; 7 Discipline of Linguistics, The University of Sydney, Sydney, Australia

**Keywords:** Psychology, Human behaviour, Evolution, Social anthropology

## Abstract

Prosociality and cooperation are key to what makes us human. But different cultural norms can shape our evolved capacities for interaction, leading to differences in social relations. How people share resources has been found to vary across cultures, particularly when stakes are high and when interactions are anonymous. Here we examine prosocial behavior among familiars (both kin and non-kin) in eight cultures on five continents, using video recordings of spontaneous requests for immediate, low-cost assistance (e.g., to pass a utensil). We find that, at the smallest scale of human interaction, prosocial behavior follows cross-culturally shared principles: requests for assistance are very frequent and mostly successful; and when people decline to give help, they normally give a reason. Although there are differences in the rates at which such requests are ignored, or require verbal acceptance, cultural variation is limited, pointing to a common foundation for everyday cooperation around the world.

## Introduction

Do people provide assistance and share resources in the same ways and to the same degrees worldwide? Or does culture cause patterns of cooperative behavior to vary in appreciable ways? Previous research, using both observational and experimental methods, has found cultural variation, especially where stakes are relatively high and where participants are anonymous. This study focuses on cooperative events that are low in stakes but high in frequency, among kin and other close familiars. We find that cultural variance is lowest at this smallest scale of social life.

We use a novel approach to describe small-scale patterns of human cooperation (called *recruitment* events), complementing and extending knowledge from existing research based on experimental games, field observation, and social-network modeling, among other methods. We thus aim to contribute to an integrated understanding of human prosocial behavior. We adopt a comparative behavioral coding approach to video-recorded data from everyday social life in diverse cultural settings. We focus on decisions made around mundane prosocial actions that are spontaneously requested, offered, and received in home and village life, as when passing someone a knife in the kitchen, taking a pot off the fire for them, or flicking a light switch for someone who cannot reach it. Our study describes and compares the structural features of these prosocial actions, including their frequency, rates of compliance, and reasons for non-compliance, and explores whether these features differ between kin and non-kin interactions. More broadly, our central question is: to what extent are everyday cooperative events organized by common principles and to what extent do they vary across human groups?

Our research is based in extensive field work and on the analysis of video recordings of everyday home/village life in a set of geographically, linguistically, and culturally diverse field sites. We examine human cooperation at the smallest scale observable, that is, in the scale of real-time social interaction in which actions (built primarily from words) are exchanged at the rate of about one every two seconds^1^. We employ a novel comparative method with high ecological validity, sampling both WEIRD (“Western, Educated, Industrialized, Rich, Democratic”) and non-WEIRD populations^2^. Because real-time social interaction is enchronic in nature—that is, it consists of structured, forward-feeding, rapid sequences of moves and counter-moves between participants^3^—patterned exchanges of actions can be defined and measured, enabling systematic comparison and quantitative analysis. The exchanges of actions in focus in our study are helping/sharing sequences in which a person produces a signal that elicits immediate assistance (e.g., says “I need a knife” or visibly struggles to reach the knife) and another person responds to that signal, typically by providing assistance.

The themes of prosociality, interdependence, reciprocity, altruism, and cooperation have been central to anthropological research based on in-depth, qualitative investigations going back more than a century^4–7^. An important line of subsequent work prioritized experimental control for systematic comparison across cultures, using economic games such as the Ultimatum Game^8^ and the Dictator Game^9^. These experimental studies found that people in diverse societies universally opt against pure selfishness when a resource can be shared, but have also shown appreciable variation in how such sharing is done^10–12^. An important feature of these economic games was that they were played with high stakes, for example to the value of one or more days’ wages^10,12^, and designed to simulate sharing/giving scenarios that are relatively infrequent in kind (e.g., sharing spoils of a hunt). Another feature was the anonymity of experiment participants, which means that social relations were not invoked in, or affected by, the decision made, and that results from these experiments do not tell us how prosocial behavior may differ, for example, between strangers versus familiars, or between kin and non-kin. Yet in many communities it is rare to interact with someone and not know who they are or how they are related to you. More recent studies have addressed this issue by developing semi-anonymous experimental designs that include recipient identities while maintaining decision confidentiality^12,13^. Further, subsequent economic-game research has sought to evaluate consistency in prosocial behavior in and out of the laboratory. While certain aspects such as the resources used to play games (e.g., money or food) do not seem to affect decision-making about allocation of resources^14^, other findings show that levels of giving may significantly differ between experimental games and observed behavior^15^. This suggests that games should be tailored to respond to, and test—but not simply recreate—real-world scenarios. An example of this is the use of experimental games to assess the impact of different motivations for sharing (e.g., reciprocity versus demand sharing), which would be difficult to achieve through observation alone^16^. Yet another feature of experimental games is that they introduce unique demand effects, that is, expectations influencing appropriate behavior created by the game itself. But proposals have been made for design principles to mitigate this^17^. Indeed, observational studies too are subject to reactive effects^18^. However, the absence of a task reduces the influence of research objectives on naturally occurring behavior, and reactive effects can be further mitigated in observational studies that rely on the researcher’s long-term participation, membership, and relationships of trust in a community, as in the case of the study reported on here. Participants in our field sites were largely unbothered by the presence of researchers and recording equipment, and did not show any evidence of changing their behavior in the kinds of mundane interactions examined here.

Another important line of work on human prosociality and cooperation has been guided by the theoretical approach of human behavioral ecology (HBE). This approach seeks to understand human behavior from an adaptive evolutionary perspective, using a range of methods that encompass observation, experiments, and social-network modeling^19–21^. Observational research has explored various dimensions of prosociality, from food-sharing^22^ to cooperative breeding^23^ to social-support networks^24^, with techniques ranging from the systematic tracking of foraging bouts to focal following to the measurement of spatial proximity using portable sensing technology^25^. Other studies have used data from everyday conversations to examine norm enforcement and reputational matters around cooperation and reciprocity, counting instances of verbal criticisms and complaints about social others^26^. Our study extends this research as well as earlier observational work in the field of human ethology^27^, which pioneered the description of verbal and nonverbal features of actions of requesting and giving in everyday home/village life^28^.

Our approach complements this previous research in three main ways. First, we focus on prosocial actions among kin and other close familiars that are low in individual stakes per instance—such as passing an item or performing a service around the house—but cumulatively high in social stakes, because the decisions are made in public, repeatedly, and with implications for reciprocity and reputation^29–32^. Second, we draw on data from video-recorded interactions of people going about their everyday lives. This method does not create goals or elicit responses, but rather observes interactions that would have occurred without our study taking place. Crucially, these interactions are captured on high-definition video and audio, enabling repeated inspection and analysis at a fine level of grain. Third, our scale of observation is at the level of moment-by-moment interaction, focusing on the interlocked steps of participants in helping/sharing sequences, rather than, say, tracking food transfers over days, weeks, or months. We apply a sequence-organizational method from microsociology^33^ that allows us to examine rapid exchanges of interactional moves and counter-moves, and that provides a structural grid for coding and comparison across languages and cultures^34,35^.

We identify and analyze over one thousand *recruitment* events, defined as helping/sharing sequences in which people produce signals that elicit immediate assistance in everyday life, in domestic and informal settings on five continents. Our corpus of video recordings features more than 350 individuals—family, friends, neighbors—representing eight diverse languages and cultures: Murrinhpatha (northern Australia), Siwu (eastern Ghana), Cha’palaa (northern Ecuador), Lao (Laos), Italian (Italy), English (UK/US), Polish (Poland), and Russian (Russia)^36^.

In the Methods, we explain how we have optimized for a balance between ecological and external validity in our data, sampling both WEIRD and non-WEIRD communities, and a comparable range of everyday activities and informal relationships.

Taken together, the findings of prior research on resource-sharing and cooperation suggest that culture should cause prosocial behavior to vary in appreciable ways. Traditional ethnographic accounts have shown the importance of local ethics^6^; economic-game research has found significant differences driven by cultural norms and values^37^; and human behavioral ecology has documented patterns of adaptation to specific natural, technological, and socio-economic environments^19^.

Moreover, kin selection theory^38,39^ predicts that interacting among kin or non-kin should have an impact on prosocial behavior, and observational research in various cultural settings has shown that relatedness between individuals increases both the frequency and degree of resource-sharing^40–43^. However, kin selection often interacts with other social and ecological factors, and in some cases has been shown to be a less powerful driver of prosocial behavior than other mechanisms such as reciprocal altruism^44^.

The results of our study are consistent with theories of human behavior that emphasize the pervasiveness of communal orientations and reciprocity across cultures and across different kinds of social relations^45–48^, and further align with theories that posit a universal infrastructure for social interaction^49–51^.

## Results

### Summary

We identify four key areas for the comparison of small-scale prosocial behavior in different cultures: i) the frequency of recruitment events; ii) responses to recruitment; iii) the formal design of compliance and rejection; and iv) the rationalization of compliance and rejection. Our main findings can be summarized as follows:

Frequency of recruitment.

- Recruitment events, where a desire or need for immediate help is signaled, are very frequent in everyday interaction among familiars, whether kin or non-kin, occurring on average once every 2.3 min.
- Variability in recruitment frequency is best explained by activity type rather than by culture: rates of recruitment are higher in *task*-focused interactions (e.g., cooking) and lower in *talk*-focused interactions (conversation for its own sake).

Responses to recruitment

- When immediate help is sought, people comply, on average, seven times more often than they decline; six times more often than they ignore; and nearly three times more often than they either decline or ignore. This preference for compliance is cross-culturally shared and unaffected by whether the interaction is among kin or non-kin.
- Although people comply six times more often than they ignore on average, we find higher tolerance of ignoring recruiting moves in at least one culture (Murrinhpatha speakers, northern Australia).

Formal design of compliance and rejection

- The asymmetry between compliance and rejection is reflected in the formal design of these moves: while the vast majority of rejections are verbalized, most complying responses, in most cultures, are produced without words.
- In Western cultures (English and Italian speakers), we find a greater tendency for people to use words when complying with recruitment, often to explicitly grant assistance before providing it.

Rationalization of compliance and rejection

- The asymmetry between compliance and rejection is further reflected in norms of rationalization: when people provide assistance, this is done without explanation, but when they decline, they normally give an explicit reason.

### Frequency of recruitment

On average in our total data set, there is one recruitment event per 2.3 minutes of continuous interaction (see Table 1). This high frequency is characteristic of the smallest-scale level of social interaction focused on here. Even the lowest overall rate we observe—less than five minutes per event—is many orders more frequent than high-stakes decisions such as sharing the spoils of a successful whale hunt or contributing to the construction of a village road^7^, the kinds of events that economic-game studies are seen to emulate.

**Table 1:** Overall frequency of recruitment.

Language	*N* interactions	Total interaction time	*N* recruitment events	Overall frequency of recruitment
Cha’palaa	14	9h15m	126	4m24s
English	16	8h21m	163	3m04s
Italian	15	3h25m	157	1m19s
Lao	12	2h46m	117	1m25s
Murrinhpatha	15	3h30m	75	2m48s
Polish	15	7h42m	173	2m40s
Russian	15	3h17m	144	1m22s
Siwu	10	3h00m	145	1m14s
TOTAL	112	41h16m	1100	
AVERAGE	14	5h09m	138	2m17s

Overall frequency of recruitment events and other characteristics of the data, including (i) the number of interactions sampled, (ii) the total interaction time sampled, and (iii) the total number of recruitment events.

**Figure 1:**
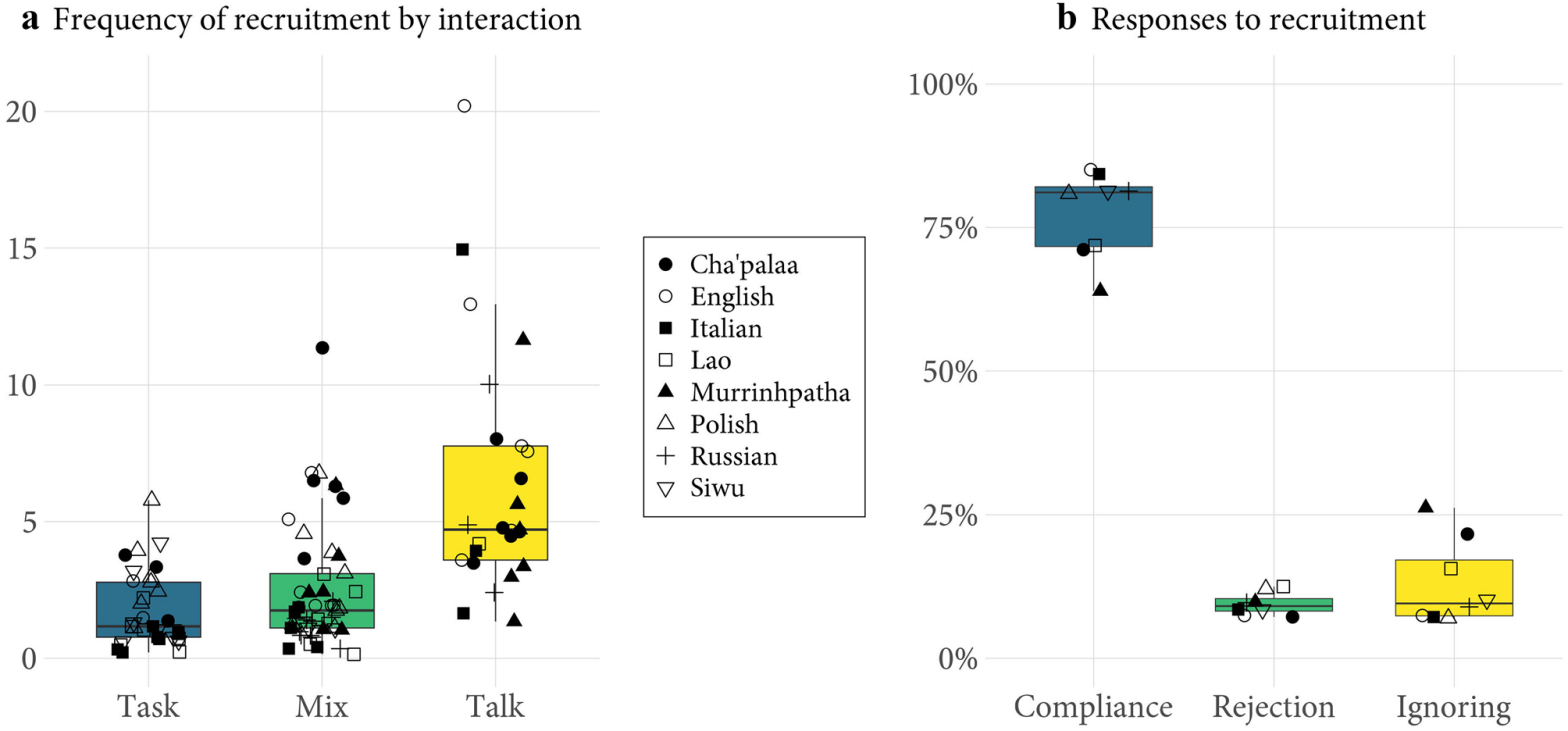
(**a**) Frequency of recruitment by interaction. Frequency of recruitment, measured in minutes, at the level of single interactions (total *n* = 109) and across activity types: task-focused, talk-focused, or a mix of talk and task. Languages are represented by different symbols. The graph excludes two Murrinhpatha interactions where no recruitment events were observed (both talk-focused, 2–3 participants). It also excludes one English interaction (talk-focused, 2 participants) with a recruitment frequency of 39.5 m to increase readability. (**b**)Responses to recruitment. Proportion of three main types of response to recruitment: the recruitee either complies (*n* = 752), rejects (*n* = 90), or ignores the recruitment (*n* = 108). Each symbol represents the proportion of a given response type in the corresponding language. Only event-final cases are counted, that is, responses that were not further followed up on.

There is variability in overall rates of recruitment across our language samples (Table 1). To assess whether this is driven by language/culture, we must consider other factors that can affect recruitment frequency at the level of single interactions. One is the number of participants in an interaction: the more participants, the more opportunities for seeking and providing assistance. A simple linear regression confirms this tendency in our total data set: the number of minutes per recruitment event decreases, on average, by 0.88 for each additional participant in an interaction (*β* − 0.88, *SE* .26,* p* = .001). This raises the possibility that differences in overall rates of recruitment (Table 1) may be affected by variability in the average number of participants per interaction across our language samples (see Table 4). Another factor that affects recruitment frequency is the type of activity that participants are engaged in: the interaction may be *talk*-focused (conversation for its own sake), *task-*focused (cooking, doing housework, repairing something together), or an interaction where talk is mixed with intermittent tasks (during meals, for example, people alternate conversation and tasks such as passing items). Figure 1a shows that recruitment frequency tends to be highest in task-focused interactions (mean 1.7m, median 1.2m), lower in mixed talk/task interactions (mean 2.5m, median 1.7m), and lowest in talk-focused interactions (mean 7.7m, median 4.7m).

When we test the effect of all three factors taken together—number of participants, activity type, and language/culture—we find that activity type is the only statistically significant predictor of recruitment frequency. Relative to the average recruitment frequency across activity types, *task*-focused interactions decreased the number of minutes per recruitment event by 1.71 (*β* − 1.71, *SE* .46,* p* < .001) and *talk*-focused interactions increased it by 3.31 (*β* 3.31, *SE* .61,* p* < .001) (see Methods, Statistical Analysis 1, for details). The finding that language/culture is *not* a statistically significant predictor of recruitment frequency is consistent with ethnographic observation: we have no evidence of cultural norms that would generally inhibit recruitment in any of the communities in our study. We thus conclude that differences in overall rates of recruitment across our language samples (Table 1) are primarily driven by the proportion of task-focused, talk-focused, and mixed talk/task interactions in each sample (see Methods, Video Corpora and Sampling).

Another question that our data set allows us to address is whether similar rates of recruitment are found in interactions among kin and in interactions among non-kin. Kin selection theory suggests that, all else being equal, helping/sharing events should occur more often between kin than between non-kin^38^. However, we find no such kin bias on rates of recruitment, neither in the aggregate nor in individual cultures (see Methods, Statistical Analysis 2).

In sum, we find that recruitment events are ubiquitous in informal interaction globally. We could theoretically imagine a culture in which people ask for assistance only once an hour, or only a few times a day, or in which people avoid asking for assistance outside or inside kin relations, but this is not what we find. Around the world, people not only ask for help, they seldom let three minutes go by without doing so while cooking, doing housework, or during meals. Only during conversation for its own sake do recruitment events become more sporadic.

### Responses to recruitment

For every recruitment event, we can ask whether the recruitment was successful, that is, whether the event resulted in the recruitee carrying out the appropriate cooperative behavior or not. Two general possibilities are *compliance* and *non-compliance*. Within non-compliance, we can further distinguish between *rejecting*, where the recruitee explicitly signals that they will not carry out the desired behavior, and *ignoring*, where the recruitee does not comply but also does not signal that they are not complying. The relative incidence of compliance versus rejection versus ignoring reveals the extent to which people are willing to help when recruited (Figure 1b).

We find that, on average, recruitees comply seven times more often than they reject, six times more often than they ignore, and nearly three times more often than they either reject or ignore recruiting moves. This pattern is robust across languages and is unaffected by whether the interaction is among kin or non-kin (see Methods, Statistical Analyses 3 and 4). However, we do find at least one culture, that of Murrinhpatha speakers of northern Australia, allowing a significantly higher tolerance of ignoring a recruitment relative to the cross-linguistic average (OR 2.98, 95% CI 1.52–5.86, *p* = .002). In an account of the social and cultural background of ignoring recruiting moves in Murrinhpatha, Blythe^52^ speculates that ignoring may in fact be a culturally evolved solution to dealing with “humbug”—pressure to comply with persistent demands for goods and services—in an Australian Aboriginal cultural context. Still, our data show that Murrinhpatha speakers regularly comply with and rarely reject recruitments (Figure 1b).

Overall, we take these findings as evidence of a pervasive cooperative stance in everyday interaction around the world. When people are prompted to, or otherwise given the opportunity to, assist others, they will usually do so, only rarely refusing, regardless of whether they are interacting among kin or non-kin. Ignoring a signal for assistance is also generally infrequent, but more common in some cultures.

### Formal design of compliance and rejection

**Figure 2:**
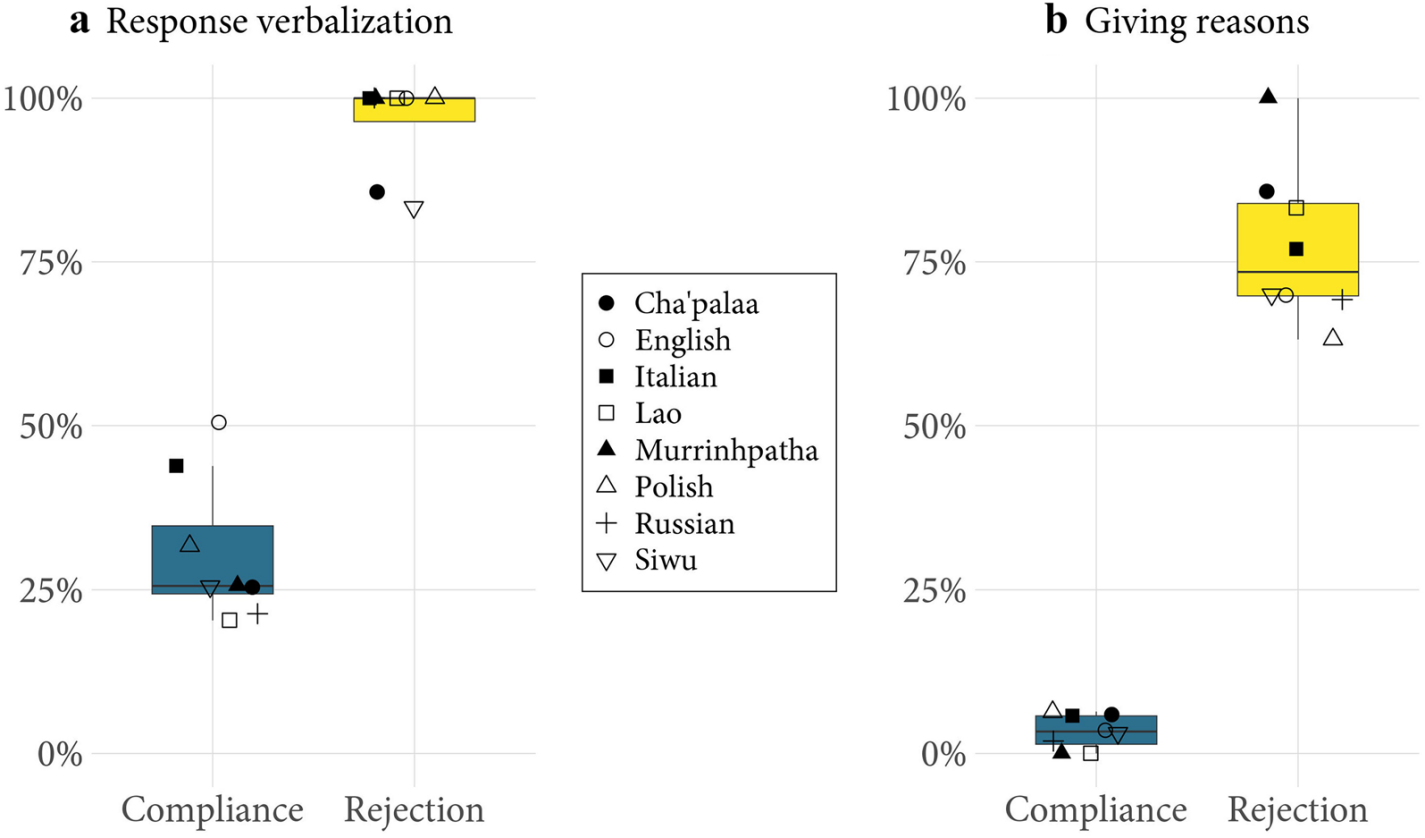
(**a**) Response verbalization. Proportion of complying (*n* = 724) and rejecting responses (*n* = 75) that involve a verbal component. Each symbol represents the proportion of verbalization for a given response type in the corresponding language. Most complying responses, in most languages, are produced without words, whereas nearly all rejections are verbalized. Only two rejections, one in Cha’palaa and one in Siwu, out of seven and six respectively, were not verbalized. Some responses could not be coded as verbal or nonverbal due to partial information in the recording, for example if the speech was inaudible. (**b**) Giving reasons. Proportion of complying (*n* = 737) and rejecting responses (*n* = 90) that are accompanied by a reason. Each symbol represents the proportion of reasons for a given response type in the corresponding language. Some responses could not be coded due to partial information in the recording, for example if the recruitee was off camera at the time of responding or if the speech was inaudible.

Compliance and rejection differ not only in frequency but also in formal design. A first feature of formal design is whether the response involves a spoken utterance (verbal) or consists only of bodily action (nonverbal). We find that, in most languages, most complying responses are produced without words, whereas rejections are almost always verbalized (Figure 2a).

While there seems to be a cross-culturally shared norm for verbalizing rejections, the design of complying responses shows some cultural variation. We find that, in English and Italian, it is significantly more common for recruitees to use words when responding to recruitment relative to the cross-linguistic average (English: OR 2.54, 95% CI 1.65–3.91, *p* < .001; Italian: OR 1.86, 95% CI 1.21–2.85, *p* = .005), an effect driven by complying responses in these languages (see Methods, Statistical Analysis 5, for details). While there is no single explanation for the verbalization of complying responses, in our earlier research on the linguistic design of recruiting moves in these languages we observed a high incidence of interrogative/question strategies (e.g., “Can you bring me a knife?”)^53,54^ relative to the cross-linguistically more frequent imperative/order strategies (e.g., “Bring me a knife”). While giving someone an order anticipates only its fulfillment, *asking* someone if they can do something makes relevant the *granting* of assistance before providing it, which is often verbalized (e.g., “Yes”, “Sure”). The relative prevalence of this pattern in Western languages can be linked to cultural differences around autonomy and responsibility in everyday cooperation^55^.

Turning to rejections, we can consider the social motivations behind their verbalization in the light of another finding. When someone declines to do what they are being recruited to do, their words will normally express a *reason* explaining *why* the action is not being done^56,57^. This is shown in Figure 2b. We find that rejections are regularly accompanied by a reason (e.g., “I’m busy”, “There’s too little left”), and that this contrasts with what people do when they comply: they seldom if ever explain why they are doing so. Moreover, although the proportion of unexplained rejections varies descriptively (Figure 2b), culture does not have a statistically significant effect on giving reasons in these data (see Methods, Statistical Analysis 6).

Further on the formal design of rejections, we find that recruitees typically decline assistance *without* saying “No” or equivalent. Whether accompanied by a reason or not, saying “No” is never found in more than one third of rejections (Table 2). The majority of rejections consist instead of simply giving a reason for non-compliance. Recruitees may use other strategies for rejection that do not involve either a giving a reason or saying “No”, for example, by telling the recruiter to assist themselves (e.g., Italian speaker: “Get your own knife!”), by questioning the recruitment (e.g., Polish speaker: “Why turn the light on?”), or by visibly refusing to cooperate (e.g., Cha’palaa speaker pulls hands away to refuse giving an object; Siwu speaker turns away to refuse taking an object). While the form and frequency of such rejections varies, our earlier research suggests that, in different cultures, unexplained rejections are disaffiliative, that is, they are associated with social dissonance or friction between recruiter and recruitee^54,58,59^.

**Table 2:** Rejection design.

Language	Reason only	“No” + reason	Just “no”	Other
Cha’palaa	.86 (6)	.0 (0)	.0 (0)	.14 (1)
English	.60 (6)	.10 (1)	.20 (2)	.10 (1)
Italian	.46 (6)	.31 (4)	.0 (0)	.23 (3)
Lao	.83 (10)	.0 (0)	.17 (2)	.0 (0)
Murrinhpatha	1.0 (6)	.0 (0)	.0 (0)	.0 (0)
Polish	.53 (10)	.11 (2)	.05 (1)	.32 (6)
Russian	.62 (8)	.08 (1)	.0 (0)	.31 (4)
Siwu	.50 (5)	.20 (2)	.10 (1)	.20 (2)
ALL	.63 (57)	.11 (10)	.07 (6)	.19 (17)

Proportion (*n*) of rejection strategies: (i) only giving a reason, (ii) saying “No” + giving a reason, (iii) just saying “No”, or (iv) conveying rejection in another way, such as visibly refusing to cooperate by turning away.

In social interaction generally, people do not ask for reasons or volunteer them unless normative social expectations have been somehow contravened^60–62^. The fact that complying responses in our data are hardly ever accompanied by explanations is compatible with the idea of a cross-culturally shared, normative expectation that people will respond cooperatively to low-cost, immediate impositions. This goes hand in hand with a shared norm for rationalizing rejection as a dispreferred response^33,56^. We also find that, when rejecting attempts to recruit assistance, people around the world tend to avoid saying “No”, often letting the rejection be inferred solely from the reason they provide for not complying.

## Discussion

Comparisons of resource-sharing decisions in fully or partly anonymous, often one-shot experimental interactions involving significant resources have found that human tendencies toward prosociality are influenced by striking cultural diversity^10–12^. These experimental findings are interpreted in the light of infrequent and high-stakes occasions for prosocial behavior in real life, from Orman (Kenya) approaches to building public infrastructure to Papua New Guinean practices around debt-inducing gift-giving, to meat-sharing among whale hunters of Lamalera (Indonesia). By contrast, in this study we have examined frequent, low-cost transactions in which social familiars share items for everyday use, perform services around the house or village, or direct one another to fulfill immediate, practical goals. Our novel method has used the endogenous structure of interactional sequences as a grid for quantitative comparison across cultures. While this reveals both similarities and differences, what is most striking is that helping/sharing behavior at the smallest scale of human interaction is evidently less susceptible to cultural variation than in more socially marked affairs.

We find cross-culturally shared principles of high entitlement to assistance, reflected in very high frequency of eliciting assistance (once every few minutes), high likelihood of compliance (occurring seven times more often than rejection), and provision of reasons when assistance is declined. These principles hold across kin and non-kin interactions (where participants are social familiars). We also find a degree of cultural variation, with some cultures showing a relatively higher tolerance of ignoring recruiting moves or a greater tendency to verbalize the provision of assistance. Our argument is that the observed variation inflects but does not fundamentally alter the organization of low-cost cooperation at the smallest scale of human interaction^34,35^. Recruitment events remain primarily shaped by low-level pressures that favor cooperation, division of labor, and reciprocity among humans^47^.

The theory of reciprocal altruism^48^ suggests that cooperation of the kind reported here—low-cost, within close and enduring relationships, and based on transparent need—should be ubiquitous, as we find. Our study supports this theory, and similar ideas such as communal sharing^45^ and generalized reciprocity^46^. It also adds to a growing literature on need-based helping among kin and other close familiars related both to reciprocity and interdependence^63,64^. We achieve this through the application of a novel method in this field (video recording and coding of rapid sequences of moves and counter-moves in everyday life) and through empirical, cross-cultural work on prosocial behavior at the smallest scale. We offer findings about real-time social interaction and about specific aspects of the formal design of recruiting and responding moves, including the range of qualitatively nuanced options that prospective helpers have at their disposal, from nonverbal compliance to verbal granting, from ignoring to explicit rejection, from giving reasons to avoiding saying “No”. We argue that these distinctions are not by-products or corollaries of reciprocal altruism but are fundamental building blocks of how cooperation is achieved by humans in the flow of everyday life.

The proposed universality of the forms of social influence that people use in helping/sharing events is supported by the fact that the patterns we observe across human groups in this study are largely unique among primates. For comparison, over 90% of food transfers in most non-human primates happen in the form of “tolerated taking” or “passive sharing”^65,66^, which is not considered prosocial behavior^67^. Resource sharing as a result of “requests” or “offers” does occur in non-human primates, with orangutans and chimpanzees showing sensitivity to explicit “signals of need”^66^, but there are few species where this regularly happens as a result of prosocial dispositions^68^. While the evolutionary roots of human altruism are a matter of ongoing debate, our finding of relatively low cultural permeability of prosocial behavior in small-scale, high-frequency, low-cost transactions is compatible with the proposal that a uniquely evolved cooperative psychology^69^ together with an enchronic infrastructure for social interaction^70,71^ provide a common foundation for all social and cultural activity^49–51,72^.

Our results derive from a focus on maximally informal interactions among social familiars, representing the most basic and primary sphere of social life^51^. We hope that this study will provide a basis for future research to extend the analysis to small-scale cooperation in other kinds of context, including interactions among strangers and interactions in institutional/formal settings, as well as to explore the ramifications of small-scale prosocial expectations, such as the longitudinal effects of deviant behavior on individuals’ reputations and relationships.

The sequences of behavior we have studied here operate at the minimal level of granularity for social coordination and alignment^73^. But they are far from minimal in importance. While the immediate individual cost of small-scale cooperative events is low, their social effect accumulates, with hundreds of such events occurring every day in a community, often repeated, and with clear implications for reciprocity and reputation^29–32^. Large-scale social realities are built out of small-scale moments like these, and it is only by studying them that we can hope to understand the foundations of human sociality.

## Methods

### Field work and communities of study

**Figure 3:**
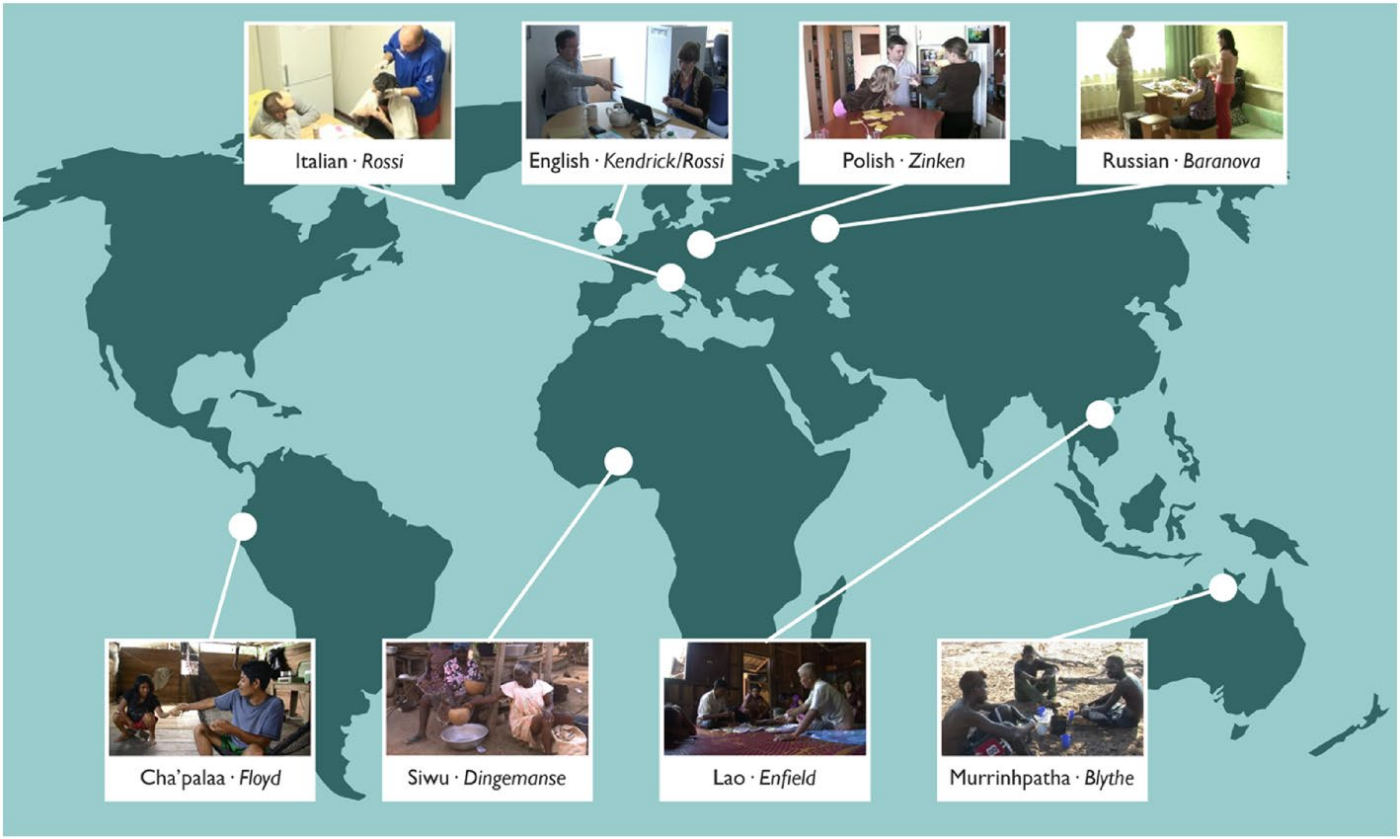
Locations of data collection. The map was created in CorelDRAW 2022 using a public-domain (CC0 1.0) vector file from Open Clipart (https://openclipart.org/detail/1733/world-map) and images from our video data.

Our research is based in extensive field work and on the analysis of video recordings of social interaction in everyday home/village life in a set of geographically, linguistically, and culturally diverse field sites (Table 3 and Figure 3).

**Table 3:** Languages.

Language	Language family	Location	Data collected by	Coding and analysis by
Cha’palaa	Barbacoan	Ecuador	Floyd	Floyd
English	IE (Germanic)	UK/US	Rossi	Kendrick
Italian	IE (Romance)	Italy	Rossi	Rossi
Lao	Tai	Laos	Enfield	Enfield
Murrinhpatha	Southern Daly	Australia	Blythe	Blythe
Polish	IE (Slavic)	Poland	Zinken	Zinken
Russian	IE (Slavic)	Russia	Baranova	Baranova
Siwu	Kwa	Ghana	Dingemanse	Dingemanse

Languages included in this study and authors responsible for data collection and analysis. (IE = Indo-European). Part of the English data were drawn from the Language and Social Interaction Archive created by Leah Wingard, available from San Francisco State University (http://www.sfsu.edu/~lsi/), and from two public-domain data sources, “Chicken Dinner” and “Virginia”.

Our field sites gave us access to both WEIRD (“Western, Educated, Industrialized, Rich, Democratic”)^2^ and non-WEIRD populations. The communities of English, Italian, Polish, and Russian speakers in our study can be considered WEIRD, while the communities of Cha’palaa, Lao, Murrinhpatha, and Siwu speakers can be considered non-WEIRD. This assessment is based on sociocultural and demographic features including education level; engagement in wage labor; reliance on outside goods, services, and technologies; reliance on subsistence farming or foraging for daily food; dominant religious practice; connection to global media; communications; and language. Measures of these features and detailed commentaries on each community are provided in the Supplementary Materials, Table S1.

All methods and protocols were carried out in accordance with relevant guidelines and regulations approved by the directorate of the Max Planck Institute for Psycholinguistics—the host institution for this project—and approved by the ethics review board of the European Research Council in the course of its compliance reviews of the ‘Human Sociality and Systems of Language Use’ project. The approved protocols align with the Max Planck Institute for Psycholinguistics’ DOBES Project Ethical and Legal Code of Conduct and Data Access Protection Rules (https://dobes.mpi.nl/dobesprogramme/ethical_legal_aspects/). Data collection was conducted with fully informed consent of participants and/or their legal guardian(s) in each field site. Informed consent was provided for participation in the study and for permission to use identifying images from the data in presentations, print, and electronic publications, including online open-access publications.

### Video corpora and sampling

Video recordings of social interaction were made in the course of building general-purpose corpora for the study of language and everyday home/village life in our field sites. For English, the general-purpose corpus was complemented with recordings from existing databases. More information about the data specific to each field site, including locations and years of data collection is provided in the Supplementary Materials. To maximize comparability, researchers ensured that the video recordings had the following properties: i) high-definition video, ii) no task given, iii) the researcher was mostly not present during the recording, iv) spontaneous interaction among non-strangers, in familiar environments.

The types of activities recorded varied from cooking together, doing housework, playing games, to just sitting together and talking. Some of the corpora contained more *task*-focused interactions (e.g., cooking), while others contained more *talk*-focused ones (conversation for its own sake), or more interactions where talk was mixed with intermittent tasks (during meals, for example, people alternate conversation and tasks such as passing items). These imbalances are due to specific field conditions and differences in the process of corpus building across sites. The researcher for Murrinhpatha (Blythe), for example, had access primarily to outdoor interactions. In this community, people prefer to spend their time away from the home, as houses are often overcrowded. This meant we had little access to domestic, task-focused activities that often take place indoors. By contrast, the Polish corpus was built with a focus on family settings and the researcher (Zinken) partnered directly with families to make recordings. This led to mostly domestic activities such as meals, cooking, and housework. As a third example, most English data were collected while the researcher (Rossi) was a visiting scholar in the UK, finding participants through a local university and local contacts, and in some cases approaching participants extempore in outdoor areas of a university campus. This corpus was complemented with recordings from existing databases to increase the number of interactions outside the university environment, including family and meal interactions, and to incorporate data from the US (see Supplementary Materials for further information).

The general-purpose corpora ranged in size from 10 to 90 hours of footage, often including dozens of interactions. In sampling these corpora for the present study, we initially identified interactions with higher clarity of sound and vision in the recording to facilitate accurate coding. For languages that the researcher did not speak natively, we prioritized interactions that had already been transcribed and translated with the collaboration of local language consultants. Then, we used three main criteria for inclusion: 1) avoid multiple interactions featuring the same individuals; 2) include interactions from different social groups (e.g., different families, different circles of friends and neighbors); 3) exclude interactions with an institutional purpose (e.g., a formal encounter between elders in a village, co-workers meeting about work rather than to socialize, service encounters in a campus cafeteria). The goal was to construct comparable samples of maximally informal interaction, representing the most basic and primary sphere of social life^51^. As shown in Table 4, this process resulted in an average sample of 14 interactions per language (range: 10-16); the number of individuals appearing across all interactions was between 34-66, for a total of 363 individuals; and the average number of participants in an interaction was 3.7 (range: 2-9). Descriptions of each interaction, including the main participants, their relationships, and basic demographics, the nature of the interaction, and the context in which the interaction took place are provided in the Supplementary Materials.

**Table 4:** Sampling.

Language	Locations	Social groups	Interactions	Individuals across all interactions	Participants per interaction: average (range)
Cha’palaa	3	14	14	39	2.8 (2–5)
English	7	15	16	51	3.4 (2–6)
Italian	6	13	15	66	4.5 (2–9)
Lao	2	7	12	36	4.6 (2–7)
Murrinhpatha	1	11	15	34	3.5 (2–4)
Polish	2	8	15	36	2.2 (2–4)
Russian	4	11	15	60	4.8 (2–9)
Siwu	1	9	10	41	4.2 (3–6)
TOTAL	26	88	112	363	
AVERAGE	3	11	14	45	3.7

Number of locations (villages, towns, or cities), social groups, and interactions sampled; total number of individuals across all sampled interactions; and average number of participants per interaction.

Wherever possible, researchers included both interactions among kin and interactions among non-kin (e.g., friends, neighbors, co-workers). Interactions involving a mix of kin and non-kin were counted separately. This process resulted in an overall cross-linguistic balance of kin and non-kin data. However, not all languages had representation of kin and non-kin interactions. More information on the sampling of kin and non-kin interactions is provided in the Supplementary Materials.

A central motivation of this study was to break new ground in achieving ecological validity in the study of human sociality at the smallest scale. We focused on examining real-time, quotidian cooperation in order to test and interpret the broader meaning of findings established in previous research based on economic games and in human behavioral ecology. Our approach naturally resulted in trade-offs between ecological validity and external validity in our data, such that we found it necessary to work with relatively modest language sample sizes, and with certain imbalances in the structure of data samples from the different languages. We were nevertheless able to ensure a wide range of speakers, settings, and activities for observation in each sample. Our statistical analyses using mixed-effects models (see Methods) take into account potential dependencies between data points by including, wherever possible, random effects at the level of the village/town where the recruitment event took place, the family or other social group represented by participants, and the particular episode of interaction that participants were engaged in. We submit that this approach has achieved a unique balance between ecological and external validity, allowing us to make robust generalizations without compromising the naturalness and richness of the data.

### Recruitment events

*Recruitment* is defined as an interactional sequence in which a person produces a signal, verbal or nonverbal, that elicits immediate assistance from another person, for example when someone asks for a knife in the kitchen, someone points to an area of the house that needs cleaning, or someone is visibly struggling to reach a light switch and another flicks it for them. A recruitment sequence canonically involves one participant—“Person A”—doing or saying something to another participant—“Person B”, such that B can see or hear it, and next, as a response, B doing something for or with A, or doing something that is otherwise relevant to the expectation of assistance. A recruitment sequence can be schematized as follows:

- **Move A:** participant A says or does something to participant B, or that B can see or hear;
- **Move B:** participant B responds in one of three main ways:
  - ***Compliance*:** doing a practical action for or with participant A that is fitted to what A has said or done;
  - ***Rejection:*** explicitly signaling unwillingness or inability to comply with the recruitment;
  - ***Ignoring:*** not complying but also not signaling non-compliance.

In addition to the three main response types—compliance, rejection, and ignoring—recruiting moves may be responded to in other ways, for example by asking for clarification (e.g., “Huh?” or “Which knife?”). Since recruitment events rarely ended with a request for clarification (*n*=5), we did not examine this response type here. We also excluded a miscellaneous set of infrequent response types (*n*=64) such as delegating the desired action to a third party or offering information that the recruiter can use to do the desired action themselves^55^.

By defining recruitment as a sequence of recognizable actions, we were able to systematically identify such events in video recordings of natural interaction in eight languages and cultures on five continents, for a total of 1100 recruitment events. A recruitment *event* may involve one or more *attempts* before being resolved. When a first attempt requires follow-up due to a problem of attention, understanding, ability, or willingness on the part of B, the sequence is expanded with additional moves.

For the sampling of recruitment events, researchers were instructed to begin collecting instances either at an arbitrary point in the video recording or from the beginning. A frequent criterion for selecting a starting point was, again, to target stretches of interaction with higher clarity of sound and vision in the recording to facilitate accurate coding. Wherever they began, researchers collected all the instances they found in a continuous stretch of interaction. They initially collected cases liberally, that is, if unsure whether something was a case, they noted it anyway and we resolved it later in research team meetings. Collection of cases in any given recorded interaction stopped either when the researcher reached 15 cases in that interaction (to avoid overrepresentation of an interaction) or when the recording ended. Child-involved cases were noted but excluded from this study. The goal was to construct samples of at least 200 adult-only recruitment sequences (attempts) per language.

### Coding

Through intensive data workshops and team analysis, we collectively surveyed the observed linguistic and behavioral practices used for initiating and responding to recruitment in different languages and developed a comparative coding scheme. The complete codebook is published under an open-access license^74^. Each researcher administered the scheme in coding the cases for their language.

Because recruitments, by definition, elicit immediate assistance, in most cases the response to a recruiting move could be observed within the subsequent few seconds in the recording. In some cases, it took longer to establish compliance, rejection, or ignoring, and if this was unresolved or unclear in the recording, the chosen coding option was “can’t tell”.

We determined that the coding scheme was consistently applied by all coders through a reliability test on a subset of the English cases, which was independently second-coded by the other seven researchers. Coding categories not meeting standards of reliability were excluded or re-coded using a narrower coding instruction. Table 5 shows two measures of inter-coder reliability—Krippendorff’s *α*^75^ and simple percent agreement (PA)—achieved by each coding category focused on in this study. Category (ii) was later fully re-coded using a narrower instruction to increase consistency. The new instruction removed a “no uptake” option and clarified the distinction between “Rejects”, “Ignores”, and “Other”^74^.

**Table 5:** Inter-coder reliability.

Coding category	Coding options	α	PA
(i) What is the sequential position of the recruitment attempt?	one and only; first of non-minimal; last of non-minimal; nth	0.80	75.6
(ii) How does the recruitee respond?	complies; rejects; ignores; initiates repair (e.g., says “Huh?” or “Which knife?”); other	0.669	65.9
(iii) Does the response include a positive polar element (e.g., “Yes”), negative polar element (e.g., “No”), or neither?	yes positive; yes negative; neither	0.87	82.9
(iv) Does the response include an explanation?	yes; no	0.72	82.5

Krippendorff’s *α* and simple percent agreement (PA) achieved by each coding category focused on in this study (before any re-coding was done).

### Statistical analyses

All statistical analyses were conducted in RStudio^76^ with R 4.1.3^77^. We used the *lme4* 1.1-30 package and its *lmer* and *glmer* functions^78^ to fit (generalized) linear mixed models^79^, and the *ggplot2* 3.3.6 package to generate graphs^80^. Wherever possible, mixed-effects models allowed us to capture dependencies within the data at the level of *locations*, social *groups*, and *interaction* episodes (random effects) while testing the main predictors of interest for recruitment behavior (fixed effects). We treated our random effects as nested: *location* → *group* → *interaction*.

Models with maximal random-effect structure often led to a “singular fit” warning and to random-effect variance estimates of zero or near-zero. We took these as indications of overfitting. To address the issue, we adopted an “iterative reduction of model complexity” approach^81^. This typically led us to remove *location* and sometimes both *location* and *group* from the random-effects structure. For Analyses 1 and 2, *interaction* was excluded as the number of levels coincided with the number of observations.

After obtaining a non-singular fit, we tested the statistical significance of full models by comparing them to null models with only the random effects^82^ using a likelihood ratio test (R function *anova*). We used a similar model-comparison approach to test the statistical significance of multiple fixed effects.

Categorical predictors were coded with sum or treatment contrasts, as indicated for each analysis. For sum contrasts, the level assigned [–1] in the contrast matrix and omitted from the comparison was the level with dependent-variable mean or proportions closest to the overall average.

An extended version of the following analyses incorporating tables and model outputs is provided in the Supplementary Materials.

**(1) *Modeling recruitment frequency as predicted by number of participants, activity type, and language.*** For this analysis, we excluded two Murrinhpatha interactions where no recruitment events were observed (both talk-focused, 2-3 participants); this left us with a total of 110 interactions. *Recruitment frequency* per interaction (in minutes) and the number of *participants* were numerical variables; *activity type* and *language* were categorical variables coded with sum contrasts. The raw recruitment frequency means and number of observations for each set of categorical contrasts are given in Table S4.

We began by fitting a maximal model with *recruitment frequency* as the dependent variable; *activity type*, *participants*, and *language* as fixed effects; and with *location* and *group* as nested random effects (intercepts). This model resulted in a “singular fit” warning and random-effect variance estimates of near-zero. We therefore reduced the random-effect structure by removing *location*, resulting in a non-singular fit and positive random-effect variance. We then compared the full model (AIC 611.16, logLik **−** 292.58) to a null model with only the random effect of *group* (AIC 630.30, logLik **−** 312.15), yielding a statistically significant difference (*χ*^2^(10) 39.14, *p* < .001). The full model (Table S5) shows statistically significant effects of *activity type* (task-focused: *β* **−** 1.57, *SE* .49, *p* = .003; talk-focused: *β* 3.06, *SE* .66, *p* < .001) and *language* (English: *β* 3.37, *SE* 1.16, *p* = .005), but not of *participants*, on *recruitment frequency*.

We then compared the full model (AIC 611.16, logLik **−** 292.58) to a reduced model without *participants* as a fixed effect (AIC 609.16, logLik **−** 292.58). As expected, the comparison between the two models was not statistically significant (*χ*^2^(1) 0, *p* = .996). We next compared the reduced model to a further reduced model without *language* as a fixed effect (607.37, logLik **−** 298.68). The comparison between the two models was not statistically significant (*χ*^2^(7) 12.21, *p* = .094). We therefore selected the simpler model (Table S6) with *activity type* as the only fixed effect as the final model (task-focused: *β* **−** 1.71, *SE* .46, *p* < .001; talk-focused: *β* 3.31, *SE* .61, *p* < .001).

**(2) *Modeling recruitment frequency as predicted by interacting among kin vs non-kin.*** For these analyses, we excluded two Murrinhpatha interactions where no recruitment events were observed (both among non-kin) and nine interactions involving a mix of kin and non-kin. This left us with a total of 101 interactions. *Recruitment frequency* per interaction (measured in minutes) was a numerical variable and interacting among *kin vs non-kin* was a dichotomous variable coded with a treatment contrast.

We began by fitting a maximal model for the total data set with *recruitment frequency* as the dependent variable; interacting among *kin vs non-kin* as a fixed effect; and with *location* and *group* as nested random effects (intercepts). The model did not result in a “singular fit” warning and was the final model (Table S7), showing that interacting among *kin vs non-kin* did not have a statistically significant effect on *recruitment frequency* (*p* = .954).

We also conducted individual analyses for each language for which we had instances of separate kin and non-kin interactions, fitting models with *recruitment frequency* as the dependent variable; interacting among *kin vs non-kin* as a fixed effect; and *location* and *group* as nested random effects (intercepts). The inclusion of *location* (for languages with two or more locations) always led to random-effect variance estimates of near-zero, so we removed the term. In two analyses, the inclusion of *group* as the only random effect also led to the same issue, so we ran simple linear regressions instead. None of these language-specific models yielded a statistically significant effect of interacting among *kin vs non-kin* on *recruitment frequency* (Tables S8-S13).

(3) ***Modeling responses to recruitment as predicted by language.*** In these analyses, we compared rates of rejection, and rates of ignoring, to rates of compliance. *Response type* was a dichotomous variable: rejecting vs complying in the first analysis and ignoring vs complying in the second; *language* was a categorical variable coded with sum contrasts. The raw response-type proportions and number of observations for each set of categorical contrasts are given in Tables S14-S15.

We began by fitting maximal models with *response type* as the dependent variable; *language* as a fixed effect; and with *location*, *group*, and *interaction* as nested random effects (intercepts). These models resulted in a “singular fit” warning and random-effect variance estimates of near-zero. We therefore reduced the random-effect structure by removing both *location* and *group* to obtain a non-singular fit. We then compared the full models (rejecting vs complying: AIC 585.86, logLik **−** 283.93; ignoring vs complying: AIC 632.34, logLik **−** 307.17) to null models with only the random effect of *interaction* (rejecting vs complying: AIC 575.55, logLik **−** 285.77; ignoring vs complying: AIC 636.56, logLik **−** 316.28). The full model for ignoring vs complying was significantly different from the corresponding null model (*χ*^2^(7) 18.22, *p* = .011), whereas the full model for rejecting vs complying was not (*χ*^2^(7) 3.68, *p* = .815). These analyses showed that *language* (Murrinhpatha) had a statistically significant effect on rates of ignoring (OR 2.98, 95% CI 1.52–5.86, *p* = .002), but not on rates of rejection, against rates of compliance (Tables S16-S17).

(4) ***Modeling responses to recruitment as predicted by interacting among kin vs non-kin.*** For these analyses, we excluded nine interactions involving a mix of kin and non-kin, corresponding to 94 recruitment events; this left us with a total of 856 recruitment events. *Response type* was a dichotomous variable: rejecting vs complying in the first analysis and ignoring vs complying in the second; interacting among *kin vs non-kin* was also a dichotomous variable coded with a treatment contrast.

We began by fitting maximal models for the total data set with *response type* as the dependent variable; interacting among *kin vs non-kin* as a fixed effect; and with *location*, *group*, and *interaction* as nested random effects (intercepts). These models resulted in a “singular fit” warning and random-effect variance estimates of near-zero. Models with both *group* and *interaction* as random effects led to the same issue, so we further reduced the random-effect structure by keeping only *interaction*. These models (Tables S18-S19) showed that interacting among *kin vs non-kin* did not have a statistically significant effect on rates of rejection (*p* = .503), nor on rates of ignoring (*p* = .491), against rates of compliance.

We also conducted individual analyses for each language for which we had instances of separate kin and non-kin interactions, fitting models with *response type* as the dependent variable; interacting among *kin vs non-kin* as a fixed effect; and *interaction* as a random effect (intercept) when it did not lead to a “singular fit” warning. None of these language-specific models yielded a statistically significant effect of interacting among *kin vs non-kin* on *response type* (Tables S20-S31).

**(5) *Modeling response verbalization as predicted by response type and language.*** In this analysis, *response verbalization* was a dichotomous variable (verbal vs nonverbal); *response type* was a dichotomous variable (compliance vs rejection); and *language* was a categorical variable. Both *response type* and *language* were coded with sum contrasts. The raw proportions of verbalized responses and number of observations for each set of categorical contrasts are given in Table S32.

We began by fitting a maximal model with *response verbalization* as the dependent variable; *response type* and *language* as fixed effects; and with *location*, *group*, and *interaction* as nested random effects (intercepts). This model resulted in a “singular fit” warning and random-effect variance estimates of near-zero. We therefore reduced the random-effect structure by removing *location*, resulting in a non-singular fit. We then compared the full model (AIC 913.19, logLik − 445.60) to a null model with only the random effects (AIC 1064.58, logLik − 529.29), yielding a statistically significant difference (*χ*^2^(8) 167.39, *p* < .001). We also compared the full model to a reduced model without *language* as a fixed effect (AIC 925.05, logLik − 458.52), yielding a statistically significant difference (*χ*^2^(7) 25.85, *p* < .001). We therefore selected the more complex model with both *response type* and *language* as fixed effects as the final model (Table S33). The model shows a statistically significant effect of both *response type* (rejection: OR 97.5, 95% CI 23.2–409.0, *p* < .001) and *language* (English: OR 2.54, 95% CI 1.65–3.91, *p* < .001; Italian: OR 1.86, 95% CI 1.21–2.85, *p* = .005) on *response verbalization*.

**(6) *Modeling giving reasons as predicted by response type and language.*** In this analysis, *giving reasons* was a dichotomous variable (reason given vs no reason given when responding to recruitment); *response type* was a dichotomous variable (compliance vs rejection); and *language* was a categorical variable. Both *response type* and *language* were coded with sum contrasts. The raw proportions of reasons given and number of observations for each set of categorical contrasts are given in Table S34.

We began by fitting a maximal model with *giving reasons* as the dependent variable; *response type* and *language* as fixed effects; and with *location*, *group*, and *interaction* as nested random effects (intercepts). This model resulted in a “singular fit” warning and random-effect variance estimates of near-zero. A model with both *group* and *interaction* as random effects led to the same issue, so we further reduced the random-effect structure by keeping only *interaction*, resulting in a non-singular fit. A comparison of the full model to a null model with only the random effect of *interaction* was not possible because the null model resulted in a singular fit. The full model (Table S35) shows that *response type* had a statistically significant effect on *giving reasons* (rejection: OR 106.3, 95% CI 42.1–268.4, *p* < .001), whereas *language* did not.

Finally, we compared the full model (AIC 355.13, logLik − 167.56) to a reduced model without *language* as a fixed effect (AIC 344.90, logLik − 169.45). The comparison between the two models was not statistically significant (*χ*^2^(7) 3.77, *p* = .806). We therefore selected the simpler model (Table S36) with *response type* as the only fixed effect as the final model (rejection: OR 106.8, 95% CI 42.1–270.7, *p* < .001).

## Supplementary Information

Supplementary Information.

## Data Availability

The primary coding data generated during this study and an R script to reproduce the statistical analyses are available in the Supplementary Materials. The original video data are not publicly available due to privacy and confidentiality reasons, but excerpts may be made available from the corresponding author upon reasonable request.
