# Supplementary material for: Shared cross-cultural principles underlie human prosocial behavior at the smallest scale
Source: Sci Rep. 2023 Apr 19;13:6057. doi: 10.1038/s41598-023-30580-5 (PMC10115833; doi:10.1038/s41598-023-30580-5)
Supplement: Supplementary file 1 — Supplementary Information. [file 41598_2023_30580_MOESM1_ESM.pdf]

Supplementary Materials for "Shared cross-cultural principles underlie human prosocial behavior at the smallest scale"

This PDF file includes:
- Supplementary Text, subdivided into:
  - Sociocultural/demographic features of WEIRD and non-WEIRD communities
  - Video corpora and descriptions of sampled interactions
  - Activity types and interactions among kin or non-kin
  - Statistical analyses with tables and model outputs
- Tables S1 to S36

Other Supplementary Materials provided as separate files include:
- Data S1 (.xlsx), subdivided into:
  - Coding data by interaction (sheet 1)
  - Coding data by recruitment attempt (sheet 2)
- Code S1 (.R)

Supplementary Text

Sociocultural/demographic features of WEIRD and non-WEIRD communities

To document the degree to which the communities examined in this study could be considered WEIRD ("Western, Educated, Industrialized, Rich, Democratic") or non-WEIRD, we collected sociocultural/demographic information relative to education level, engagement in wage labor, reliance on outside goods/services/technologies, reliance on subsistence farming or foraging for daily food, dominant religious practice, connection to global media, communications, and language. Each researcher provided detailed commentaries on these sociocultural/demographic features by responding to the following set of questions about their community of study, based on deep ethnographic familiarity with the culture, grounded in long-term field work (and community membership, in some cases).

Q1. Formal education. What level of formal education, if any, did people generally have? Formal education is intended as education that is relatively standardized and delivered by trained teachers.
Q2. Global economy. To what extent did the community participate in the global economy?
Q2a. Wage labor. To what extent did community members engage in wage labor? Wage labor is when a worker "sells" his or her labor to an employer for a specified weekly wage or monthly salary, typically on terms and conditions determined by the employer.
Q2b. Outside goods/services/tech. To what extent did the community rely on goods, services, or technology produced in other countries, especially industrialized ones?
Q2c. Farming/foraging for food. To what extent did the community rely on subsistence farming or foraging for food?
Q3. Religion. What religion(s), if any, did people subscribe to?
Q4. Telecommunications. What proportion of the community had access to the Internet and/or telephone?

Q5. Fluency in a European language. What proportion of the community was fluent in a large, European language?

The questions were answered with respect to the communities of study at the time of data collection, which in many cases dates back more than a decade. This is important because there have been significant changes over that time; most notably, the rapid changes in accessibility of Internet and cell phones in several of these communities.

Table S1 summarizes the sociocultural/demographic information about each community in the form of approximate measures (e.g., ranging from none-low-mid-high-all). These measures are based on the more detailed commentaries reported after the table.

	**WEIRD**				**non-WEIRD**			
**Language spoken**	**English**	**Italian**	**Polish**	**Russian**	**Cha'palaa**	**Lao**	**Murrinhpatha**	**Siwu**
Formal education, older adults	All high school, many university	All high school, many university	All high school, many university	All high school	None	Low	Low	Low
Formal education, younger adults	All high school, many university	All high school, many university	All high school, many university	All high school, a few university	Grade school, some high school	Grade school, some high school	Grade school	Grade school, some high school
Amount of wage labor	High	High	High	High	Low	Low	Mid	Low
Reliance on outside goods, services, tech	High	High	High	High	Mid	Mid	High	Mid
Reliance on subsistence farming or foraging for food	None	None	None	Mid	High	High	Mid	High
Dominant religion	Christian (Anglican, Catholic)	Christian (Catholic)	Christian (Catholic)	Christian (Orthodox)	Indigenous practice, with Catholic elements	Buddhism, with Indigenous practice elements	Christian (Catholic), with Indigenous practice elements	Christian (assorted)
Access to phones or Internet	High	High	High	High	None	None	None	None
Fluency in a large, European language	Native	Native	Native	Native	Low	None	Low	Mid

Table S1. Sociocultural/demographic features of the communities examined: WEIRD and non-WEIRD.

Cha'palaa speakers in Chachi communities of northern Ecuador (researcher: Simeon Floyd)
Q1. Formal education. It has historically been rare for the Chachi people to receive formal education, and most of the older members of the community (over 50) had no school experience, while younger adults may have completed a few years of grade school, and in rare cases may have attended some high school. Children and teenagers currently receive more schooling than previous generations and an increasing percentage of young people complete high school. Only a

few Chachis have attended university, including one participant in these recordings (in CHSF2012_01_07S1).
Q2a. Wage labor. Only a small percentage of men in the community engaged in wage labor outside of the local area, otherwise the other men and almost all women work in local non-waged activities like farming and fishing.
Q2b. Outside goods/services/tech. Traditional houses and other goods like baskets, boats, and tools are made locally with native materials, but in recent decades commerce for goods from outside the area has increased to some extent.
Q2c. Farming/foraging for food. Chachi subsistence is largely based on local agriculture, foraging, fishing, and hunting, with limited livestock raising. During recent decades there has been some increase in sale of local products like wood and cash crops, but this is still a relatively minor part of subsistence.
Q3. Religion. While a brief period of missionization in the 1500s and 1600s lead to the incorporation of some Roman Catholic elements in Chachi religion, Chachis maintain an elaborate indigenous ritual calendar with several large religious events per year that represent strong continuities with pre-Colombian religion. So, while it has adopted some Christian symbols and concepts, Chachi religion is still largely indigenous.
Q4. Telecommunications (Internet/phones). At the time the recordings were made (2008-2013) there was no usage of telephones or internet in most Chachi communities, with a few community members beginning to have access to basic cellular phones that could only be used in cities outside of the Chachi communities, with the majority not using them at all. By 2022, particularly in connection with online education during the COVID pandemic, internet connections have reached more communities, meaning that more people are now familiar with smartphones than at the time of recording, but mainly younger community members.
Q5. Fluency in a European language. A minority of speakers of Cha'palaa are able to speak Spanish, the national language of Ecuador, limited mainly to adult men who have had some experience working outside of the community. Other adults may have some limited command of Spanish but could not be considered fluent. Older people, especially women, and young children are almost all monolingual in Cha'palaa; high school age children current are starting to learn more Spanish than the previous generation.

English speakers in the United Kingdom and United States (researcher: Giovanni Rossi)
Q1. Formal education. Participants in many recordings were students at a large university in the UK (York), mostly undergraduate, a few graduate. Other participants in the corpus had at least a high-school diploma and none had less than high-school education.
Q2a. Wage labor. Most participants who were not students engaged in wage labor, and some students did too.
Q2b. Outside goods/services/tech. All the locations where the data were collected were highly integrated in the global economy including for goods, services, and technology.
Q2c. Farming/foraging for food. None.
Q3. Religion. Ethnographic information on religion is limited. However, the dominant religion in the areas of data collection was Christianity, including Anglican and Catholic denominations.
Q4. Telecommunications (Internet/phones). Virtually everyone had access to both phones and the Internet with the exception of participants in the pre-Internet recordings "Chicken Dinner" and "Virginia".

Q5. Fluency in a European language. All participants were speakers of English. Ethnographic information on fluency in other languages is limited. However, it is likely that some of the student participants had some competence, especially receptive, in other European languages including French, Spanish, and German.

Italian speakers in Italy (researcher: Giovanni Rossi)
Q1. Formal education. Most participants had a high-school diploma (12-13 years of schooling), many had a university degree (16-17 years of schooling), and only a minority had less than high-school education. Formal education in Italy is highly institutionalized and organized at both national and regional levels.
Q2a. Wage labor. Most participants who were not students engaged in wage labor, and some students did too. A few participants were self-employed professionals.
Q2b. Outside goods/services/tech. All participants lived in northern Italy, a highly industrialized and economically developed area, with lots of goods, services, and technology coming from other European countries as well as from all around the world.
Q2c. Farming/foraging for food. Reliance on subsistence farming or foraging was very rare, except for a few individuals who owned land or lived in mountain areas and partially relied on garden farming.
Q3. Religion. The vast majority of people subscribed to Roman Catholicism, with most people middle-aged and older attending religious services weekly. The holiday house that appears in the recordings was run by a local parish.
Q4. Telecommunications (Internet/phones). Virtually everyone had access to the Internet and phones, except a few elderly people who had a landline but no Internet.
Q5. Fluency in a European language. Most participants were effectively Italian monolinguals. Some had receptive competence in English, German, or French. Few participants had active competence in these or other languages.

Lao speakers in Laos (researcher: N. J. Enfield)
Q1. Formal education. Participants ranged from primary school education (older participants) to high school education, all at village level. National education is in the Lao language.
Q2a. Wage labor. Vast majority of participants did not engage in wage labor; they were rice farmers and small-scale merchants (e.g., selling vegetables at market).
Q2b. Outside goods/services/tech. Many goods were produced in industrialized countries, such as refrigerators, televisions, motor vehicles, hand tools, personal care products, etc. At the same time, many goods, services, and products were home-made, such as the houses people live in, their furniture, mats, etc.
Q2c. Farming/foraging for food. Heavy reliance on subsistence farming, mostly rice farming, and also garden/orchard farming; all raise some livestock including chickens, cattle, pigs.
Q3. Religion. Theravada Buddhism was the dominant religion; all sizeable villages (including those appearing in these recordings) had at least one temple, with resident monks.
Q4. Telecommunications (Internet/phones). At the time of these data recordings (2001, 2002, 2003, and 2011) the rate of ownership of (smart) phones, and access to the Internet, was close to zero.
Q5. Fluency in a European language. All but one of the participants were effectively monolingual, though all had good receptive competence in Thai, the language of neighboring Thailand, which is in a dialect relationship with Lao (i.e., practically speaking, Lao and Thai are

not separate languages, but dialects, they are mutually intelligible). Lao speakers know Thai through exposure to Thai media, television shows, and music. Only one participant in these recordings had functional competence in English. No other European languages are known by people appearing in these recordings (no French or Russian).

Murrinhpatha speakers in northern Australia (researcher: Joe Blythe)
Q1. Formal education. The Murrinhpatha speakers in this collection are mostly monolingual. Some have received some primary education in the bilingual school program (English), although this has been sporadic as school retention rates are exceedingly low. Most participants have very low levels of literacy and numeracy.
Q2a. Wage labor. The older participants are all pensioners. The younger participants are either unemployed or work for four hours a day in the Community Development Employment Program.
Q2b. Outside goods/services/tech. Houses are overcrowded and largely devoid of furniture, except for mattresses and the occasional chair. Cooking is either conducted outdoors on an open fire or indoors on an electric stove or in an electric frying pan. Most houses contain a television but virtually nobody owns a computer. Some households have cars.
Q2c. Farming/foraging for food. Community members rely heavily on food from the shop that is trucked in from cities. They supplement this diet by foraging for vegetables and hunting for game, especially on the weekends. Traditional foods are highly valued.
Q3. Religion. The religion is predominantly Roman Catholic. The variety of Catholicism is not regarded as incompatible with traditional Indigenous Australian creation belief systems centering around the performative actions of totemic ancestors.
Q4. Telecommunications (Internet/phones). When the data were collected (2007-2012), Internet access was virtually nil. Mobile phones began appearing around 2012 but these were not smartphones. Landlines existed in a few households, but most people relied on public telephones.
Q5. Fluency in a European language. Most participants are largely monolingual, although many have passive knowledge of English. Some elder participants have knowledge of other endangered traditional languages (i.e., Marri Amu, Marri Tjevin, Marri Ngarr) but did not speak these languages regularly.

Polish speakers in Poland (researcher: Jörg Zinken)
Q1. Formal education. All participants had formal school education for at least 9 years, many had a high-school diploma (13 years of schooling) and a university degree (16-17 years). Formal education is highly institutionalized and centralized at the national level.
Q2a. Wage labor. Most participants who were not children were engaged in wage labor. A few participants were retired.
Q2b. Outside goods/services/tech. All participants lived in university cities, most in the South-East of Poland, some in central-eastern Poland — both highly industrialized and economically developed areas. Many goods, services, and technology come from other European countries as well as from all around the world.
Q2c. Farming/foraging for food. Virtually no reliance on subsistence farming or foraging. Some participants grow fruit and vegetables, keep chicken, or pick mushrooms recreationally.
Q3. Religion. Virtually all participants subscribed to Roman Catholicism. Some only attend church for major religious holidays, while in some families, religious practices are part of everyday life (saying grace, children preparing for first Holy Communion).

Q4. Telecommunications (Internet/phones). Virtually everyone had some form of Internet access, however, connectivity was bad at the time of recording, and using the Internet was not yet part of everyday life for most people. Everyone had landline telephone connections.
Q5. Fluency in a European language. Most participants were effectively Polish monolinguals, though some had receptive competence in English, German, or Russian. Few participants had active competence in these or other languages.

Russian speakers in Russia (researcher: Julija Baranova)
Q1. Formal education. The majority of participants had 9 to 11 years of schooling. Many had about 3 years of additional vocational education. A small number had a university degree, and two participants had a doctoral degree. Formal education in Russia is highly institutionalized and organized at both the national and regional level.
Q2a. Wage labor. Most participants engaged in wage labor. A few participants were self-employed professionals; some were retired.
Q2b. Outside goods/services/tech. Many goods, services and technology that people used in their daily lives were produced either from industrial sources in Russia or in China. Western products and services were also available in principle but more expensive and often not accessible to participants in this sample.
Q2c. Farming/foraging for food. Although all necessary food products were available from stores, many participants had their own gardens where they grew vegetables and fruit, some of which they canned and stored for the winter. Many participants relied on their gardens for food.
Q3. Religion. Most participants belong to the Russian Orthodox Church. Several participants were of Tatar origin and were possibly Muslim. There was at least one Orthodox church in the location where the recordings were made. Most participants, however, did not attend services on a regular basis but rather for main celebrations, such as Christmas, Easter, etc.
Q4. Telecommunications (Internet/phones). All participants had access to phones and most of them had a cell phone. Most elderly participants (70+) had no access to the Internet, but the rest generally did, either directly in their homes or through others in the community.
Q5. Fluency in a European language. Most participants were Russian monolinguals. Although English or German is taught in school, most participants do not speak or understand them. Some participants were of Tatar origin, but it was not possible to ascertain if they grew up speaking the Tatar language or were fluent in it.

Siwu speakers in eastern Ghana (researcher: Mark Dingemanse)
Q1. Formal education. Education levels ranged from no schooling (some of the elderly participants, particularly women), to primary school education (2-8 years of schooling) to secondary school education (8-15 years of schooling), all at village level. Education is in the Ewe language (first years of primary) and Ewe and English (later years of primary, all years of secondary).
Q2a. Wage labor. The vast majority of participants were engaged in day-to-day farming (cocoa, rice, maize) and small-scale merchandise (vegetables, palm oil, baskets, tools). Very few if any engaged in wage labor.
Q2b. Outside goods/services/tech. Some goods and technologies produced in industrialized countries were common (e.g., metal and plastic kitchenware, bikes, radios, TV sets, plastic chairs, knives), but also many goods, services and products were locally made, such as clay houses, wooden furniture, fireplaces, pestle & mortar, brooms, drums.

Q2c. Farming/foraging for food. Heavy reliance on subsistence farming, mostly local brown rice, cassava, yam, plantain, and maize, as well as livestock animals like chickens and goats. Foraging was limited to berries, trapping of rodents and some hunting of smaller wild ungulates.
Q3. Religion. Christianity was the dominant religion, with over 10 denominations (from Catholic, Presbyterian to various varieties of Pentecostalism) represented in the village. At the same time, faith in traditional deities connected to water sources, rivers, mountains, and other key landmarks continues to play a strong role.
Q4. Telecommunications (Internet/phones). At the time that most of the recordings were made (2007-2011) the rate of Internet access was practically zero, with no landlines in this part of Ghana and very unreliable mobile reception. Only from 2012 onward did the first mobile transmission tower in the area enable better mobile reception and low-bandwidth Internet access.
Q5. Fluency in a European language. The language of everyday communication and the native language of all participants was Siwu. Virtually all participants additionally knew some Ewe, a regional language of wider communication also used in markets, in primary education and in some churches. Participants younger than 40 additionally spoke Ghanaian English (used in post-primary education) and/or Pidgin English picked up from travelling merchants. A few elderly people also knew Akan, a language that used to have political significance in the area up to the 1960s.

Video corpora and descriptions of sampled interactions

Here we provide more information about our video corpora, including locations, years, and procedures of data collection specific to each field site. We also provide descriptions of each sampled interaction, including the main participants, their relationships, and basic demographics, the nature of the interaction, and the context in which the interaction took place.

Cha'palaa (researcher: Simeon Floyd)
Data were collected by the researcher from three villages in Chachi communities of north-western Ecuador, mostly in the Rio Cayapas area, particularly from its tributary the Rio Zapallo, between 2007 and 2015. The researcher had established long-term relationships in these communities as part of his ongoing linguistic/anthropological field research.

CHSF2011_01_11S2: A family including parents, children and grandmother eat, rest, and do household tasks at home.
CHSF2011_01_11S: Adult members of a family speak with neighbors at home.
CHSF2011_02_14S3: A family including parents and children rest and converse at home.
CHSF2011_02_15S4: A couple and their small son rest and take part in household activities like cleaning and changing clothes.
CHSF2011_06_24S3: A mother weaves baskets and converses with her daughter at home.
CHSF2011_06_25S2: A mother and daughter converse at home in a bedroom.
CHSF2012_01_07S1: An extended family rests and converses at home.
CHSF2012_01_07S3: A mother and her teenage and young daughter converse, clean house, and play at home
CHSF2012_01_20S1: A grandmother, her daughter-in-law, and several children cook, clean and converse at home.
CHSF2012_01_20S6: A middle-aged couple rests and converses at home with their small son.

CHSF2012_01_21S3: A young couple converses, rests, and does household tasks at home.
CHSF2012_08_04S3: Several women (changing as some come and go) who are both neighbors and family wash clothes and converse on the beach.
CHSF2012_08_04S4: Several women (changing as some come and go) who are both neighbors and family wash clothes and converse on the beach.
CHSF2012_08_05S5: A father and several adult and young children rest, eat, and do household tasks at home.

English (researcher: Giovanni Rossi)
Data in interactions labelled with “RCE” (for “Rossi Corpus of English”) were collected by the researcher in three urban centers in northern England (Birmingham, Sheffield, York) in 2011; the researcher secured participants via a local university and local contacts; some participants were approached extempore on a university campus. The Rossi Corpus of English is a general-purpose corpus for the study of language in social interaction. Additional interactions were sourced from data collected in the United States (including the Language and Social Interaction Archive created by Leah Wingard at San Francisco State University, http://www.sfsu.edu/~lsi/) and made available to researchers in conversation analysis. These included three interactions (BBQ, Monopoly Boys, Sunday Lunch with Family) from central and north-west US (2000s) and two (Virginia, Chicken Dinner) from south-east and south-west US (1960s/1970s).

RCE01 Cigarette: Two young women sitting down, talking, and smoking on the lawn of a university campus.
RCE02 TwoFriends: Two friends sitting down and talking on the lawn of a university campus.
RCE06 Grass: A group of students, many of them roommates, sitting down, talking, and sunbathing on the lawn of a university campus.
RCE07 Duck: Three young men sitting down and talking in front of a university building.
RCE08 UKHousemates I: Three housemates chatting, eating, and cooking in the kitchen.
RCE09 UKHousemates II: Three housemates chatting, eating, and cooking in the kitchen.
RCE14 Colleagues: Two university teachers, colleagues and friends, having tea and cookies, and talking in the office.
RCE15 Swimmers: Three young men sitting down and talking in the courtyard of a university cafeteria.
RCE22 HumStudents: A group of students sitting down and talking in the lobby area of a university building.
RCE26 Catan; Three friends playing a game of Settlers of Catan in the kitchen.
RCE28 Lake: Two young women sitting down and talking near a pond on a university campus.
BBQ: A group of friends cooking and eating in a park.
Chicken Dinner: Two young couples having dinner in the living room of an urban home.
Monopoly Boys: Two young men playing a game of Monopoly.
Sunday Lunch with Family: Family members (middle-aged mother and father, and two daughters) having lunch at home.
Virginia: Family members (middle-aged mother, a son in his 20s, his fiancée, and two teenaged daughters) having dinner at home.

Italian (researcher: Giovanni Rossi)

Data were collected in two urban areas (Bologna, Trento) and surrounding rural communities in northern Italy in the period 2009-2013. The researcher, a native of the region, utilized an extensive network of existing relationships to secure participants.

AlbertoniPrep: A middle-aged mother (50s) and her daughter (20s) engaged in food preparation together in the kitchen of an urban home.
Aldo&Bino: Two young men (20s), friends, sitting down and talking in the living room of an urban home.
BiscottiPome01: Three siblings and the girlfriend of one of them (20s) sitting down, talking, and eating leftovers in an urban home.
CampFamPrep: Adults of various ages (young, middle-aged, elderly) engaged in food preparation and tidying up together in the common kitchen area of a suburban winter holiday house.
Tinta: Three friends (20s) talking while one friend styles another's hair in the living area of a student dormitory.
MaraniPranzo: Family members (middle-aged parents, two young-adult children in their 20s, and a child's boyfriend) having lunch in the living room of an urban home.
Reparto02: Middle-aged co-workers in a healthcare setting tidying up the workplace after work.
DopoProve10: Members of a vocal ensemble (young-adult to middle-aged, mix of family and friends) sitting down, talking, and eating after music rehearsals.
Diego&Anna: A young couple (20s) sitting down and talking in the bedroom of an urban home.
Circolo01: Four retirees (in their 60s) playing cards in a residential living center.
CampUniPictionary01: A group of friends (20s/30s) engaged in food preparation in the common kitchen area of a suburban winter holiday house.
Capodanno01: Family members (teenaged to middle-aged) engaged in food preparation for a large gathering in an urban home.
Camillo; Family members (teenaged to middle-aged) engaged in food preparation for a large gathering in an urban home.
Fratelli01: Two brothers (40s) having coffee and chatting after lunch with a young child present and occasionally interacting with them.
MasoShanghai: A group of friends (20s/30s) eating, drinking, and intermittently playing a tabletop game in the living area of a suburban winter holiday house.

Lao (researcher: N. J. Enfield)
Data were collected over several years (2001-2003 and 2011) in villages in the northern district of Vientiane Municipality, Laos, where the researcher has conducted regular and sustained field research since 1990. The researcher secured access and permissions through established relationships in the field site.

INTCN_111204t: Two young mothers (20s) and a grandmother (60s) chatting in the living area of a village home (engaged in childcare).
INTCN_111203l: Husband and wife (30s) in a village home, on a kitchen verandah, engaged in food preparation (cleaning a catch of small fish).
INTCN_111202s: Young adults (20s) sitting down, talking, and eating, on a break from work in rice fields, with some children and a grandfather also present (60s).

INTCN_030731b: Family members (middle aged parents and young-adult children) engaged in food preparation together on the kitchen verandah of a village home.

INTCN_020727a: An older couple (60s) on a house visit to the village home of an elderly couple (80s) after the elderly man had been injured in a fall; middle-aged man also present.

INTCN_030806e: A middle-aged man (40s) and elderly man (70s) sitting in a village house living area, talking while they wait for food to be served; some interaction with a young (teenage) woman who is preparing food.

INTCN_111204q: Household family members (adult children, 30s, parents, 50s, grandmother, 70s) and a young woman neighbor (20s), talking as they prepare meat to grill for sale out front of the village family home.

INTCN_111204x: Family members (older parents and middle-aged/young-adult children) in the living area of the family home, talking as they sort clothes and prepare rattan for basketry.

INTCN_111201k: Late night evening in living area of household, older parents (50s/60s) and adult children (20s) chatting as they lie down and rest at home before bed.

INTCN_111202n: Village temple, young monks, a middle-aged building contractor, and a village elder (70s) chat as the monks are eating lunch.

CONV_010714b: Middle-aged parents (30s and 50s) and daughter (teenage) in the compound, preparing saplings to be planted.

CONV_020723b: Family members (middle aged parents and young-adult children) engaged in food preparation together at village home on the kitchen verandah.

Murrinhpatha (researcher: Joe Blythe)

Data were collected by the researcher and a research associate (John Mansfield) between 2007 and 2012 in the communities of Wadeye and on the estates of local clan groups in Murrinhpatha-speaking areas within the Fitzmaurice and Moyle Rivers region of Australia's Northern Territory. The researcher had established long-term relationships in these communities as part of his ongoing linguistic field research.

20070728 Teasing: Four senior women, all close friends, sitting on the floor of a community building, having just eaten lunch.

20090707 Museum: Two middle aged sisters with the teenage daughter of the elder sister. Two are sitting on the floor of a community building. One is sitting on a chair. They are waiting for their lunch to be delivered.

20091121 Da_Ngarne: Three elderly women with one of their middle-aged daughters. They are having a picnic in the bush. One woman's great-grand daughter is present.

20100814 Da_Ngurrert: Three elderly women with one of their great-grand daughters. They are having a picnic in the bush.

20100827 New_Album: Four men in their forties, all musicians, conversing on their verandah of a house. One man's mother occasionally appears.

20110730 Dingalngu: Four elderly women are having a picnic in the bush.

20110824 Bullet_Thuykem: Four young men having a cup of tea on top of a hill overlooking the community.

20110828 Nanthak: Three elderly women with one of their middle-aged daughters. They are having a picnic on the beach, in the bush.

20110901 Kreator_Thuykem: Three young men, all brothers, are having a cup of tea on top of a hill overlooking the community.
20120709 Spidi_Ngandimeli: Three young men are having a picnic on the beach.
20120715 Ngandimeli: Three young men are having a picnic in the bush.
20120602 PDW: Three young men, all close friends, sitting on the floor of a community building, having a cup of tea.
20120612 SD: Two middle-aged men, close friends, sitting on chairs in a community building, having a cup of tea.
20120711 Pulampa: Four young men, all friends, are sitting in a park in the community.
20120719 BSR: Three young men, all friends, are sitting on the ground at the barge landing, at the edge of the community.

Polish (researcher: Jörg Zinken)
Data were collected in two urban centers (Lublin, Warsaw) in central and eastern Poland. The corpus was built with a focus on family settings and the researcher partnered directly with families to make recordings between 2009 and 2012.

*PP1-1***:** A couple in their 60s, their adult daughter, and four young foster children (aged 1-5) are having lunch in the kitchen of the family home.
*PP2-1***:** A couple in their thirties and two of their children (aged 6-10) are having dinner in their living room.
*PP3-2***:** A couple in their 50s and their two children (aged 8-11) are having dinner in the living room of their house.
*PP5-4***:** A couple in their thirties and their three children (aged 1-11) are having lunch in the kitchen of their home.
*PP6-1***:** A couple in their thirties and their two children (aged 4-7) are doing a craft activity at the kitchen table in their flat.
*Pa02Apr2012***:** Two sisters (late 30s and early 40s), the elder sister's daughter (aged 8) and the younger sister's three children (aged 1-4) are in the elder sister's flat, the children are painting Easter eggs, the women are chatting and doing household work.
*MiBrApr2012***:** A couple in their thirties, two of their children (aged 1), and the woman's parents are in the parents' flat, preparing and eating breakfast in the kitchen.
*PP4-1***:** A couple in their thirties and their two children (aged 6-8) are preparing and having lunch in the family's house.
*PP2-2***:** A couple in their thirties and their three children (aged 1-10) are having supper in their living room.
*PP3-1***:** A couple in their 50s and their two children (aged 8-11) are preparing supper in the kitchen of their home.
*PP5-1***:** A couple in their thirties and their three children (aged 1-11) are having lunch in the kitchen of their home.
*PP5-5***:** A couple in their thirties and their three children (aged 1-11) are having lunch in the living room of their home.
*PP6-3***:** A couple in their thirties and their two children (aged 4-7) are playing a game at the kitchen table in their flat.
*PP4-2***:** A couple in their thirties and their two children (aged 6-8) are preparing and having lunch in the family's house.

*PP2-5***:** A couple in their thirties and two of their children (aged 6-10) are having dinner in their kitchen.

Russian (researcher: Julija Baranova)

Data were collected in rural communities in the region of Chelyabinsk in central Russia in 2011-2012. The researcher had family ties in the area and tapped into local networks of relationships to secure participants.

20110804_Colleagues_celebration: A group of nurses who are co-workers and friends, of ages ranging between about 30-55 and one 70 years old, gathered to celebrate a nurse's birthday

20110807_Family_evening: A woman and her daughter-in-law (in her 20s) sitting down and talking in the woman's kitchen.

20110826_Old_friends_A: Several girlfriends and the husband of one of them, former classmates now in their 50s, gathered for food and drinks in the living room of one of the women.

20110827_Family: A woman and her two daughters (in their 20s) having tea in the kitchen, talking, and cooking.

20120114_memorial: Family members (both close and extended family) gathered for a memorial dinner.

20120602_family_friends: A married couple (30s) are having two friends and co-workers, as well as the husband's sister, over for a visit.

20120202_cooking: Family members are gathered in the house of an older couple (in their 60s). The husband's sister and her husband are visiting, and so is their daughter (30s) with her husband and children.

20120120_colleagues_casual: School custodians are having lunch in a dedicated room in the school; some are coming and going; others are making soup, eating, and talking. They are all women aged 50-60.

20110821_Family_dinner_Country_A: In a small, remote village, an elderly couple (70s) is having family members over from another town including the woman's sister (also in her 70s), that sister's son and daughter-in-law.

20110826_Old_friends_B: Former classmates, all women, gathered in the kitchen of one of them. Two of them are twin sisters.

20110813_School_Friends: Several girlfriends (50s), former classmates, are visiting at the place of one of them, who lives with her elderly mother.

20110817_Family_dinner_B: Family members gathered in the living room of a couple (in their 50-60s). Also present are the couple's children (in their 20-30s) with their partners, and future in-laws.

20110816_Sisters_A: Three sisters (in their 50s) gathered at the place of one of the sisters, having food and drinks. One sister's husband is also present.

20110817_Niece: A woman (in her 50s) is having her niece (20s) over. They are talking in the kitchen and the woman is cooking.

20120602_Granddaughter: A woman is visiting her daughter (in her 30s) and granddaughter at their home.

Siwu (researcher: Mark Dingemanse)

Data were collected in the village of Akpafu-Mempeasem, north of Hohoe in Ghana's Volta region, over the period 2007-2013. The researcher made these recordings of everyday home/village interactions between family and friends in the course of building a general-purpose corpus for the study of language and social interaction in Siwu.

Neighbours: A family of 6 (3 adults aged 30-50, three teenagers) chatting in the outdoors living area of their compound house while shelling maize.

Compound4: Four elderly people (60s-70s) sitting in the shade of a compound house and talking while preparing food. Some interaction with a male relative (60s) passing by.

Cooking1: Household family members (adult children, 20s; mother, 40s; grandmother, 60s) talking outdoors as they shell maize, peel cassava, do the washing and various other domestic tasks.

Kitchen1: Household family members (grandmother, 60s; mother, 30s; sister, 30s, and baby) talking in the outdoors cooking area as they do washing and prepare food.

Compound5: Four friends (20s) talking in an outdoors compound area while one of them does the hair of another. Two children of one of them are also present.

Maize1: A group of 4 adults (ages 40-60) chatting while shelling maize in the outdoors area bordering their compound houses.

Tailor: Friends (30s) chatting in an outdoors area where one of them works as a tailor and is mending clothes.

Maize3: Three women (40s,60s) and a young woman neighbor (20s) talking as they shell maize, peel cassava, and prepare food while sitting outside in the shade of the house.

Palmoil1: Friends (30s) talking in the shade of a compound house. Three of them take turns pressing/extracting palm oil together.

Compound: Female relatives (sisters, 40s, aunts, 60s) sitting down to talk in an outdoors area. The sampled part is a mostly dyadic interaction between two of the aunts.

Activity types and interactions among kin or non-kin

Activity types. The types of activities recorded varied from cooking together, doing housework, playing games, to just sitting together and talking. As shown in Table S2, some of the corpora contained more *task*-focused interactions (e.g., cooking together), while others contained more *talk*-focused ones (conversation for its own sake), or more interactions where talk was mixed with intermittent tasks (during meals, for example, people alternate conversation and tasks such as passing items). These imbalances are due to specific field conditions and differences in the process of corpus building across sites. The researcher for Murrinhpatha (Blythe), for example, had access primarily to outdoor interactions. In this community, people prefer to spend their time away from the home, as houses are often overcrowded. This meant we had little access to domestic, task-focused activities that often take place indoors. By contrast, the Polish corpus was built with a focus on family settings and the researcher (Zinken) partnered directly with families to make recordings. This led to mostly domestic activities such as meals, cooking, and housework. As a third example, most English data were collected while the researcher (Rossi) was a visiting scholar in the UK, finding participants through a local university and local contacts, and in some cases approaching participants extempore in outdoor areas of a university campus. This corpus was complemented with recordings from existing databases to increase the

number of interactions outside the university environment, including family and meal interactions, and to incorporate data from the US (see above).

Language	Task-focused interactions	Mixed talk/task interactions	Talk-focused interactions
Cha'palaa	.21 (3)	.36 (5)	.43 (6)
English	.12 (2)	.44 (7)	.44 (7)
Italian	.47 (7)	.33 (5)	.20 (3)
Lao	.50 (6)	.42 (5)	.08 (1)
Murrinhpatha	.0 (0)	.47 (7)	.53 (8)
Polish	.47 (7)	.53 (8)	.0 (0)
Russian	.07 (1)	.73 (11)	.20 (3)
Siwu	.70 (7)	.30 (3)	.0 (0)
**ALL**	**.29 (33)**	**.46 (51)**	**.25 (28)**

Table S2. Proportion (*n*) of task-focused, talk-focused, and mixed talk/task interactions across language samples.

Interactions among kin or non-kin. Wherever possible, researchers included both interactions among kin and interactions among non-kin (e.g., friends, neighbors, co-workers). Interactions involving a mix of kin and non-kin were counted separately. As Table S3 shows, the process resulted in an overall cross-linguistic balance of kin and non-kin data with a slight skewing toward kin. However, not all languages had representation of kin or non-kin interactions. For Cha'palaa and Polish, the larger corpora from which the samples were drawn contained almost exclusively kin interactions, with no separate non-kin interactions. For English and Murrinhpatha, on the other hand, researchers had access primarily to non-kin interactions. That said, for languages where most interactions were among kin, we ensured that a good number of different families was sampled (Cha'palaa: 12 kin interactions and 12 families; Polish: 15 kin interactions and 8 families). For languages where most interactions were among non-kin, we ensured that a good number of different groups of friends, neighbors, or co-workers was sampled (English: 14 non-kin interactions and 13 non-kin groups; Murrinhpatha: 12 non-kin interactions and 8 non-kin groups).

Language	Kin interactions	Non-kin interactions	Mixed interactions	Families	Non-kin groups
Cha'palaa	12 (.86)	0 (.0)	2 (.14)	12	2
English	2 (.12)	14 (.88)	0 (.0)	2	13
Italian	6 (.40)	8 (.53)	1 (.07)	5	8
Lao	7 (.58)	4 (.33)	1 (.08)	2	5
Murrinhpatha	3 (.20)	12 (.80)	0 (.0)	3	8
Polish	15 (1.0)	0 (.0)	0 (.0)	8	0
Russian	9 (.60)	5 (.33)	1 (0.7)	8	3
Siwu	4 (.40)	2 (.20)	4 (.40)	5	4
**TOTAL**	**58 (.52)**	**45 (.40)**	**9 (.08)**	**45**	**43**
**AVERAGE**	**7**	**6**	**1**	**6**	**5**

Table S3. Number (proportion) of kin, non-kin, and mixed (both kin and non-kin) interactions, families, and non-kin groups.

Statistical analyses with tables and model outputs

(1) ***Modeling recruitment frequency as predicted by number of participants, activity type, and language.*** For this analysis, we excluded two Murrinhpatha interactions where no recruitment events were observed (both talk-focused, 2-3 participants); this left us with a total of 110 interactions. *Recruitment frequency* per interaction (in minutes) and the number of *participants* were numerical variables; *activity type* and *language* were categorical variables coded with sum contrasts. The raw recruitment frequency means and number of observations for each set of categorical contrasts are given in Table S4.

	**Recruitment frequency**	***N* observations**
**Activity type**		
Task	1.75	33
Mix of talk and task	2.50	51
Talk	7.70	26
**Language**		
English	7.68	16
Cha'palaa	5.29	14
Murrinhpatha	3.67	13
Polish	3.02	15
Russian	2.09	15
Italian	2.07	15
Siwu	1.53	10
Lao	1.47	12

Table S4. Raw recruitment frequency means and number of observations for each set of categorical contrasts in the analysis of recruitment frequency as predicted by number of participants, activity type, and language.

We began by fitting a maximal model with *recruitment frequency* as the dependent variable; *activity type*, *participants*, and *language* as fixed effects; and with *location* and *group* as nested random effects (intercepts). This model resulted in a "singular fit" warning and random-effect variance estimates of near-zero. We therefore reduced the random-effect structure by removing *location*, resulting in a non-singular fit and positive random-effect variance. We then compared the full model (AIC 611.16, logLik -292.58) to a null model with only the random effect of *group* (AIC 630.30, logLik -312.15), yielding a statistically significant difference ($\chi^2(10)$ 39.14, $p < .001$). The full model (Table S5) shows statistically significant effects of *activity type* (task-focused: β -1.57, *SE* .49, $p = .003$; talk-focused: β 3.06, *SE* .66, $p < .001$) and *language* (English: β 3.37, *SE* 1.16, $p = .005$), but not of *participants*, on *recruitment frequency*.

**Fixed effects**	***β***	***SE***	***t***	***p***
(intercept)	3.778	0.881	4.289	0.000 ***
activity type: task	-1.568	0.493	-3.18	0.003 **
activity type: talk	3.062	0.656	4.665	0.000 ***

participants	0.008	0.194	0.042	0.967
language: Cha'palaa	1.274	1.264	1.008	0.317
language: English	3.372	1.158	2.912	0.005 **
language: Italian	-1.125	1.216	-0.926	0.358
language: Lao	-0.671	1.826	-0.368	0.714
language: Polish	0.453	1.513	0.299	0.765
language: Russian	-1.109	1.305	-0.85	0.398
language: Siwu	-0.966	1.44	-0.671	0.505
**Random effects**	**Variance**	***SD***		
group	18.486	4.299		
Residual	2.085	1.444		
(observations = 110; *groups* = 83)				

Table S5. Full linear mixed model for the analysis of recruitment frequency as predicted by number of participants, activity type, and language.

We then compared the full model (AIC 611.16, logLik -292.58) to a reduced model without *participants* as a fixed effect (AIC 609.16, logLik -292.58). As expected, the comparison between the two models was not statistically significant ($\chi^2(1)$ 0, $p = .996$). We next compared the reduced model to a further reduced model without *language* as a fixed effect (607.37, logLik -298.68). The comparison between the two models was not statistically significant ($\chi^2(7)$ 12.21, $p = .094$). We therefore selected the simpler model (Table S6) with *activity type* as the only fixed effect as the final model (task-focused: β -1.71, *SE* .46, $p < .001$; talk-focused: β 3.31, *SE* .61, $p < .001$).

**Fixed effects**	***β***	***SE***	***t***	***p***
(intercept)	4.039	0.512	7.885	0.000 ***
activity type: task	-1.713	0.463	-3.703	0.000 ***
activity type: talk	3.313	0.607	5.462	0.000 ***
**Random effects**	**Variance**	***SD***		
group	19.493	4.415		
Residual	2.061	1.436		
(observations = 110; *groups* = 83)				

Table S6. Final linear mixed model for the analysis of recruitment frequency as predicted by number of participants, activity type, and language.

(2) *Modeling recruitment frequency as predicted by interacting among kin vs non-kin.* For these analyses, we excluded two Murrinhpatha interactions where no recruitment events were observed (both among non-kin) and nine interactions involving a mix of kin and non-kin. This left us with a total of 101 interactions. *Recruitment frequency* per interaction (measured in minutes) was a numerical variable and interacting among *kin vs non-kin* was a dichotomous variable coded with a treatment contrast.

We began by fitting a maximal model for the total data set with *recruitment frequency* as the dependent variable; interacting among *kin vs non-kin* as a fixed effect; and with *location* and

group as nested random effects (intercepts). The model did not result in a "singular fit" warning and was the final model (Table S7), showing that interacting among *kin vs non-kin* did not have a statistically significant effect on *recruitment frequency* (*p* = .954).

**Fixed effects**	***β***	***SE***	***t***	***p***
(intercept)	3.594	0.951	3.781	0.001 ***
kin vs non-kin: non-kin	0.075	1.297	0.058	0.954
**Random effects**	**Variance**	***SD***		
group	22.859	4.781		
location	3.876	1.969		
Residual	2.510	1.584		
(observations = 101; *groups* = 78; *locations* = 24)				

Table S7. Linear mixed model for the analysis of recruitment frequency as predicted by interacting among kin vs non-kin.

We also conducted individual analyses for each language for which we had instances of separate kin and non-kin interactions, fitting models with *recruitment frequency* as the dependent variable; interacting among *kin vs non-kin* as a fixed effect; and *location* and *group* as nested random effects (intercepts). The inclusion of *location* (for languages with two or more locations) always led to random-effect variance estimates of near-zero, so we removed the term. In two analyses, the inclusion of *group* as the only random effect also led to the same issue, so we ran simple linear regressions instead. None of these language-specific models yielded a statistically significant effect of interacting among *kin vs non-kin* on *recruitment frequency* (Tables S8-S13).

	β	***SE***	***t***	***p***
(intercept)	1.904	7.073	0.269	0.792
kin vs non-kin: non-kin	6.605	7.562	0.873	0.397

Table S8. English-specific linear model for the analysis of recruitment frequency as predicted by interacting among kin vs non-kin.

**Fixed effects**	***β***	***SE***	***t***	***p***
(intercept)	3.619	1.763	2.053	0.065 .
kin vs non-kin: non-kin	-2.331	2.248	-1.037	0.322
**Random effects**	**Variance**	***SD***		
group	15.544	3.942		
Residual	0.002	0.048		
(observations = 14; *groups* = 13)				

Table S9. Italian-specific linear mixed model for the analysis of recruitment frequency as predicted by interacting among kin vs non-kin.

	β	***SE***	***t***	***p***
(intercept)	1.434	0.507	2.83	0.02 *
kin vs non-kin: non-kin	0.34	0.841	0.404	0.696

Table S10. Lao-specific linear model for the analysis of recruitment frequency as predicted by interacting among kin vs non-kin.

**Fixed effects**	***β***	***SE***	***t***	***p***
(intercept)	3.01	1.83	1.644	0.142
kin vs non-kin: non-kin	0.992	2.142	0.463	0.66
**Random effects**	**Variance**	***SD***		
group	3.898	1.974		
Residual	6.153	2.480		
(observations = 13; *groups* = 10)				

Table S11. Murrinhpatha-specific linear mixed model for the analysis of recruitment frequency as predicted by interacting among kin vs non-kin.

**Fixed effects**	***β***	***SE***	***t***	***p***
(intercept)	3.095	1.107	2.796	0.023 *
kin vs non-kin: non-kin	-2.017	2.02	-0.998	0.347
**Random effects**	**Variance**	***SD***		
group	8.507	2.917		
Residual	0.077	0.277		
(observations = 14; *groups* = 10)				

Table S12. Russian-specific linear mixed model for the analysis of recruitment frequency as predicted by interacting among kin vs non-kin.

**Fixed effects**	***β***	***SE***	***t***	***p***
(intercept)	1.968	0.795	2.476	0.086 .
kin vs non-kin: non-kin	-1.039	1.273	-0.816	0.47
**Random effects**	**Variance**	***SD***		
group	1.515	1.231		
Residual	0.465	0.682		
(observations = 6; *groups* = 5)				

Table S13. Siwu-specific linear mixed model for the analysis of recruitment frequency as predicted by interacting among kin vs non-kin.

(3) ***Modeling responses to recruitment as predicted by language.*** In these analyses, we compared rates of rejection, and rates of ignoring, to rates of compliance. *Response type* was a dichotomous variable: rejecting vs complying in the first analysis and ignoring vs complying in the second; *language* was a categorical variable coded with sum contrasts. The raw response-type proportions and number of observations for each set of categorical contrasts are given in Tables S14-S15.

**Language**	**Proportion (*n*) of rejecting responses**	***N* observations**
Lao	.148 (12)	81

Murrinhpatha	.133 (6)	45
Polish	.130 (19)	146
Russian	.107 (13)	122
Siwu	.094 (10)	106
Cha'palaa	.092 (7)	76
Italian	.091 (13)	142
English	.081 (10)	124

Table S14. Raw response-type proportions and number of observations in the analysis of rejecting vs complying responses as predicted by language.

**Language**	**Proportion (*n*) of ignoring responses**	***N* observations**
Murrinhpatha	.291 (16)	55
Cha'palaa	.233 (21)	90
Lao	.179 (15)	84
Siwu	.111 (12)	108
Russian	.099 (12)	121
English	.081 (10)	124
Polish	.080 (11)	138
Italian	.079 (11)	140

Table S15. Raw response-type proportions and number of observations in the analysis of ignoring vs complying responses as predicted by language.

We began by fitting maximal models with *response type* as the dependent variable; *language* as a fixed effect; and with *location*, *group*, and *interaction* as nested random effects (intercepts). These models resulted in a "singular fit" warning and random-effect variance estimates of near-zero. We therefore reduced the random-effect structure by removing both *location* and *group* to obtain a non-singular fit. We then compared the full models (rejecting vs complying: AIC 585.86, logLik -283.93; ignoring vs complying: AIC 632.34, logLik -307.17) to null models with only the random effect of *interaction* (rejecting vs complying: AIC 575.55, logLik -285.77; ignoring vs complying: AIC 636.56, logLik -316.28). The full model for ignoring vs complying was significantly different from the corresponding null model ($\chi^2(7)$ 18.22, p = .011), whereas the full model for rejecting vs complying was not ($\chi^2(7)$ 3.68, p = .815). These analyses showed that *language* (Murrinhpatha) had a statistically significant effect on rates of ignoring (OR 2.98, 95% CI 1.52–5.86, p = .002), but not on rates of rejection, against rates of compliance (Tables S16-S17).

**Fixed effects**	**Log odds**	***SE***	***z***	***p***
(intercept)	-2.167	0.147	-14.729	0.000 ***
language: Cha'palaa	-0.178	0.383	-0.463	0.643
language: English	-0.31	0.333	-0.933	0.351
language: Italian	-0.201	0.299	-0.67	0.503
language: Lao	0.387	0.325	1.191	0.234
language: Murrinhpatha	0.222	0.422	0.528	0.598

language: Polish	0.232	0.268	0.868	0.385
language: Siwu	-0.157	0.337	-0.465	0.642
**Random effects**	**Variance**	***SD***		
interaction	0.151	0.389		
(observations = 842; *interactions* = 107)				

Table S16. Generalized linear mixed model for the analysis of rejecting vs complying responses as predicted by language.

**Fixed effects**	**Log odds**	***SE***	***z***	***p***
(intercept)	-2.033	0.145	-13.984	0.000 ***
language: Cha'palaa	0.609	0.317	1.921	0.055 .
language: English	-0.488	0.362	-1.348	0.178
language: Italian	-0.582	0.339	-1.715	0.086 .
language: Lao	0.397	0.342	1.163	0.245
language: Murrinhpatha	1.093	0.345	3.168	0.002 **
language: Polish	-0.554	0.342	-1.62	0.105
language: Russian	-0.291	0.335	-0.868	0.386
**Random effects**	**Variance**	***SD***		
interaction	0.378	0.615		
(observations = 860; *interactions* = 107)				

Table S17. Generalized linear mixed model for the analysis of ignoring vs complying responses as predicted by language.

(4) ***Modeling responses to recruitment as predicted by interacting among kin vs non-kin.*** For these analyses, we excluded nine interactions involving a mix of kin and non-kin, corresponding to 94 recruitment events; this left us with a total of 856 recruitment events. *Response type* was a dichotomous variable: rejecting vs complying in the first analysis and ignoring vs complying in the second; interacting among *kin vs non-kin* was also a dichotomous variable coded with a treatment contrast.

We began by fitting maximal models for the total data set with *response type* as the dependent variable; interacting among *kin vs non-kin* as a fixed effect; and with *location*, *group*, and *interaction* as nested random effects (intercepts). These models resulted in a "singular fit" warning and random-effect variance estimates of near-zero. Models with both *group* and *interaction* as random effects led to the same issue, so we further reduced the random-effect structure by keeping only *interaction*. These models (Tables S18-S19) showed that interacting among *kin vs non-kin* did not have a statistically significant effect on rates of rejection (p = .503), nor on rates of ignoring (p = .491), against rates of compliance.

**Fixed effects**	**Log odds**	***SE***	***z***	***p***
(intercept)	-2.162	0.18	-11.998	0.000 ***
kin vs non-kin: non-kin	-0.176	0.262	-0.67	0.503
**Random effects**	**Variance**	***SD***		
interaction	0.102	0.319		

(observations = 769; *interactions* = 98)

Table S18. Generalized linear mixed model for the analysis of rejecting vs complying responses as predicted by interacting among kin vs non-kin.

**Fixed effects**	**Log odds**	***SE***	***z***	***p***
(intercept)	-2.277	0.213	-10.696	0.000 ***
kin vs non-kin: non-kin	0.198	0.288	0.688	0.491
**Random effects**	**Variance**	***SD***		
interaction	0.406	0.637		
(observations = 779; *interactions* = 98)				

Table S19. Generalized linear mixed model for the analysis of ignoring vs complying responses as predicted by interacting among kin vs non-kin.

We also conducted individual analyses for each language for which we had instances of separate kin and non-kin interactions, fitting models with *response type* as the dependent variable; interacting among *kin vs non-kin* as a fixed effect; and *interaction* as a random effect (intercept) when it did not lead to a “singular fit” warning. None of these language-specific models yielded a statistically significant effect of interacting among *kin vs non-kin* on *response type* (Tables S20-S31).

**Fixed effects**	**Log odds**	***SE***	***z***	***p***
(intercept)	-3.520	1.301	-2.706	0.007 **
kin vs non-kin: non-kin	1.063	1.321	0.805	0.421
**Random effects**	**Variance**	***SD***		
interaction	0.704	0.839		
(observations = 124; *interactions* = 15)				

Table S20. English-specific generalized linear mixed model for the analysis of rejecting vs complying responses as predicted by interacting among kin vs non-kin.

	**Log odds**	***SE***	***z***	***p***
(intercept)	-2.73	0.596	-4.582	0.000 ***
kin vs non-kin: non-kin	0.651	0.693	0.939	0.348

Table S21. Italian-specific generalized linear model for the analysis of rejecting vs complying responses as predicted by interacting among kin vs non-kin.

	**Log odds**	***SE***	***z***	***p***
(intercept)	-1.74	0.343	-5.077	0.000 ***
kin vs non-kin: non-kin	-0.051	1.133	-0.045	0.964

Table S22. Lao-specific generalized linear model for the analysis of rejecting vs complying responses as predicted by interacting among kin vs non-kin.

	**Log odds**	***SE***	***z***	***p***

(intercept)	-18.57	2174.21	-0.009	0.993
kin vs non-kin: non-kin	16.96	2174.21	0.008	0.994

Table S23. Murrinhpatha-specific generalized linear model for the analysis of rejecting vs complying responses as predicted by interacting among kin vs non-kin.

**Fixed effects**	**Log odds**	***SE***	***z***	***p***
(intercept)	-1.612	0.391	-4.121	0.000
kin vs non-kin: non-kin	-1.36	0.727	-1.871	0.061 .
**Random effects**	**Variance**	***SD***		
interaction	0.112	0.334		
(observations = 118; *interactions* = 14)				

Table S24. Russian-specific generalized linear mixed model for the analysis of rejecting vs complying responses as predicted by interacting among kin vs non-kin.

	**Log odds**	***SE***	***z***	***p***
(intercept)	-3.497	1.015	-3.445	0.000 ***
kin vs non-kin: non-kin	-18.07	5524.412	-0.003	0.997

Table S25. Siwu-specific generalized linear model for the analysis of rejecting vs complying responses as predicted by interacting among kin vs non-kin.

**Fixed effects**	**Log odds**	***SE***	***z***	***p***
(intercept)	-3.567	1.342	-2.658	0.008 **
kin vs non-kin: non-kin	1.115	1.365	0.817	0.414
**Random effects**	**Variance**	***SD***		
interaction	0.845	0.919		
(observations = 124; *interactions* = 14)				

Table S26. English-specific generalized linear mixed model for the analysis of ignoring vs complying responses as predicted by interacting among kin vs non-kin.

**Fixed effects**	**Log odds**	***SE***	***z***	***p***
(intercept)	-2.276	0.557	-4.082	0.000 ***
kin vs non-kin: non-kin	-0.458	0.701	-0.653	0.513
**Random effects**	**Variance**	***SD***		
interaction	0.146	0.382		
(observations = 128; *interactions* = 14)				

Table S27. Italian-specific generalized linear mixed model for the analysis of ignoring vs complying responses as predicted by interacting among kin vs non-kin.

**Fixed effects**	**Log odds**	***SE***	***z***	***p***
(intercept)	-2.265	0.691	-3.276	0.001 **
kin vs non-kin: non-kin	1.812	0.997	1.818	0.069 .
**Random effects**	**Variance**	***SD***		

interaction	0.458	0.677
(observations = 75; *interactions* = 11)		

Table S28. Lao-specific generalized linear mixed model for the analysis of ignoring vs complying responses as predicted by interacting among kin vs non-kin.

	**Log odds**	***SE***	***z***	***p***
(intercept)	-1.099	0.667	-1.648	0.099 .
kin vs non-kin: non-kin	0.262	0.745	0.352	0.725

Table S29. Murrinhpatha-specific generalized linear model for the analysis of ignoring vs complying responses as predicted by interacting among kin vs non-kin.

**Fixed effects**	**Log odds**	***SE***	***z***	***p***
(intercept)	-1.814	0.459	-3.948	0.000 ***
kin vs non-kin: non-kin	-1.115	0.707	-1.577	0.115
**Random effects**	**Variance**	***SD***		
interaction	0.004	0.065		
(observations = 116; *interactions* = 14)				

Table S30. Russian-specific generalized linear mixed model for the analysis of ignoring vs complying responses as predicted by interacting among kin vs non-kin.

	**Log odds**	***SE***	***z***	***p***
(intercept)	-2.110	0.529	-3.986	0.000 ***
kin vs non-kin: non-kin	-0.123	0.806	-0.153	0.878

Table S31. Siwu-specific generalized linear model for the analysis of ignoring vs complying responses as predicted by interacting among kin vs non-kin.

(5) ***Modeling response verbalization as predicted by response type and language.*** In this analysis, *response verbalization* was a dichotomous variable (verbal vs nonverbal); *response type* was a dichotomous variable (compliance vs rejection); and *language* was a categorical variable. Both *response type* and *language* were coded with sum contrasts. The raw proportions of verbalized responses and number of observations for each set of categorical contrasts are given in Table S32.

	**Proportion (*n*) of verbalized responses**	***N* observations**
**Response type**		
Compliance	.325 (235)	724
Rejection	.973 (73)	75
**Language**		
English	.538 (64)	119
Italian	.485 (65)	134
Polish	.404 (57)	141
Murrinhpatha	.356 (16)	45

Cha'palaa	.311 (23)	74
Russian	.296 (34)	115
Siwu	.290 (29)	100
Lao	.282 (20)	71

Table S32. Raw proportions of verbalized responses and number of observations for each set of categorical contrasts in the analysis of response verbalization as predicted by response type and language.

We began by fitting a maximal model with *response verbalization* as the dependent variable; *response type* and *language* as fixed effects; and with *location*, *group*, and *interaction* as nested random effects (intercepts). This model resulted in a "singular fit" warning and random-effect variance estimates of near-zero. We therefore reduced the random-effect structure by removing *location*, resulting in a non-singular fit. We then compared the full model (AIC 913.19, logLik -445.60) to a null model with only the random effects (AIC 1064.58, logLik -529.29), yielding a statistically significant difference ($\chi^2(8)$ 167.39, $p < .001$). We also compared the full model to a reduced model without *language* as a fixed effect (AIC 925.05, logLik -458.52), yielding a statistically significant difference ($\chi^2(7)$ 25.85, $p < .001$). We therefore selected the more complex model with both *response type* and *language* as fixed effects as the final model (Table S33). The model shows a statistically significant effect of both *response type* (rejection: OR 97.5, 95% CI 23.2–409.0, $p < .001$) and *language* (English: OR 2.54, 95% CI 1.65–3.91, $p < .001$; Italian: OR 1.86, 95% CI 1.21–2.85, $p = .005$) on *response verbalization*.

**Fixed effects**	**Log odds**	***SE***	***z***	***p***
(intercept)	-0.87	0.101	-8.596	0.000 ***
response type: rejection	4.58	0.732	6.261	0.000 ***
language: Cha'palaa	-0.286	0.282	-1.015	0.31
language: English	0.932	0.22	4.246	0.000 ***
language: Italian	0.619	0.218	2.831	0.005 **
language: Lao	-0.519	0.336	-1.547	0.122
language: Murrinhpatha	-0.182	0.346	-0.525	0.6
language: Russian	-0.402	0.266	-1.513	0.13
language: Siwu	-0.263	0.252	-1.042	0.297
**Random effects**	**Variance**	***SD***		
interaction	0.064	0.254		
group	0.040	0.199		
(observations = 799; *interactions* = 106; *groups* = 82)				

Table S33. Final generalized linear mixed model for the analysis of response verbalization as predicted by response type and language.

(6) ***Modeling giving reasons as predicted by response type and language.*** In this analysis, *giving reasons* was a dichotomous variable (reason given vs no reason given when responding to recruitment); *response type* was a dichotomous variable (compliance vs rejection); and *language* was a categorical variable. Both *response type* and *language* were coded with sum contrasts. The raw proportions of reasons given and number of observations for each set of categorical contrasts are given in Table S34.

	**Proportion (*n*) of reasons given**	***N* observations**
**Response type**		
Compliance	.038 (28)	737
Rejection	.744 (67)	90
**Language**		
Murrinhpatha	.140 (6)	43
Polish	.139 (20)	144
Cha'palaa	.133 (10)	75
Italian	.125 (17)	136
Lao	.123 (10)	81
Siwu	.095 (10)	105
Russian	.092 (11)	120
English	.089 (11)	123

Table S34. Raw proportions of reasons given and number of observations for each set of categorical contrasts in the analysis of giving reasons as predicted by response type and language.

We began by fitting a maximal model with *giving reasons* as the dependent variable; *response type* and *language* as fixed effects; and with *location*, *group*, and *interaction* as nested random effects (intercepts). This model resulted in a "singular fit" warning and random-effect variance estimates of near-zero. A model with both *group* and *interaction* as random effects led to the same issue, so we further reduced the random-effect structure by keeping only *interaction*, resulting in a non-singular fit. A comparison of the full model to a null model with only the random effect of *interaction* was not possible because the null model resulted in a singular fit. The full model (Table S35) shows that *response type* had a statistically significant effect on *giving reasons* (rejection: OR 106.3, 95% CI 42.1–268.4, $p < .001$), whereas *language* did not.

**Fixed effects**	**Log odds**	***SE***	***z***	***p***
(intercept)	-3.438	0.294	-11.697	0.000 ***
response type: rejection	4.666	0.473	9.869	0.000 ***
language: Cha'palaa	0.59	0.471	1.253	0.21
language: English	-0.087	0.441	-0.198	0.843
language: Italian	0.38	0.386	0.986	0.324
language: Murrinhpatha	0.018	0.634	0.029	0.977
language: Polish	0.106	0.381	0.279	0.781
language: Russian	-0.468	0.453	-1.033	0.302
language: Siwu	-0.16	0.475	-0.338	0.735
**Random effects**	**Variance**	***SD***		
interaction	0.325	0.570		
(observations = 827; *interactions* = 106)				

Table S35. Full generalized linear mixed model for the analysis of giving reasons as predicted by response type and language.

Finally, we compared the full model (AIC 355.13, logLik -167.56) to a reduced model without *language* as a fixed effect (AIC 344.90, logLik -169.45). The comparison between the two models was not statistically significant ($\chi^2(7)$ 3.77, p = .806). We therefore selected the simpler model (Table S36) with *response type* as the only fixed effect as the final model (rejection: OR 106.8, 95% CI 42.1–270.7, p < .001).

**Fixed effects**	**Log odds**	***SE***	***z***	***p***
(intercept)	-3.442	0.288	-11.929	0.000 ***
response type: rejection	4.671	0.475	9.839	0.000 ***
**Random effects**	**Variance**	***SD***		
interaction	0.449	0.670		
(observations = 827; *interactions* = 106)				

Table S36. Final generalized linear mixed model for the analysis of giving reasons as predicted by response type and language.
